# Self-assembled wide bandgap nanocoatings enabled outstanding dielectric characteristics in the sandwich-like structure polymer composites

**DOI:** 10.1186/s40580-022-00346-2

**Published:** 2022-12-09

**Authors:** Tian-Yu Wang, Xiao-Fen Li, Shu-Ming Liu, Bai-Xin Liu, Xi-Dong Liang, Shunning Li, Gui-Xin Zhang, Jian-Bo Liu, Zhi-Min Dang

**Affiliations:** 1 State Key Laboratory of Control and Simulation of Power System and Generation Equipment, Department of Electrical Engineering, Tsinghua University, Beijing, 100084 China; 2 Key Laboratory of Advanced Materials (MOE), School of Materials Science and Engineering, Tsinghua University, Beijing, 100084 China; 3 School of Advanced Materials, Shenzhen Graduate School, Peking University, Shenzhen, 518055 China

**Keywords:** Polymer dielectrics, Self-assembled nanocoatings, Wide bandgap, Nanodomain trap parameters, Dielectric properties

## Abstract

Polymer dielectrics are insulators or energy storage materials widely used in electrical and electronic devices. Polymer dielectrics are needed with outstanding dielectric characteristics than current technologies. In this study, the self-assembly of boron nitride nanosheets (BNNSs) was applied to form an inorganic–organic nanocoating on various common polymer dielectrics. It is inexpensive and easy to fabricate this thin coating on a large scale. The coating has a wide bandgap and thus can significantly improve the breakdown strength of polymer dielectrics. The charge characteristics and trapping parameters of nano-domains on the surfaces of polymer dielectrics were measured, and the coating had shallow trap levels. This facilitated the dissipation of surface charges and thus greatly increased the flashover voltage. The coating also effectively improved the temperature stability and dielectric constant of the polymer dielectric. This nanocoating shows potential as a method to effectively improve the dielectric characteristics of polymer dielectrics and outperform existing composite polymer dielectrics, which are crucial for large-scale applications in energy storage and power and electronic devices.

**Supplementary Information:**

The online version contains supplementary material available at 10.1186/s40580-022-00346-2.

## Introduction

As good insulating or energy storage materials, polymer dielectrics are widely used in advanced electrical devices [1, 2], flexible electronic devices [3, 4] and energy storage capacitors [5–8]. However, the development trends of integration and high power of electronic and electrical equipment have put forward higher requirements on the electrical strength and high-temperature performance of polymer dielectrics as an insulating part [1, 9]. In addition, when polymer dielectrics are used as energy storage materials, the energy storage density is proportional to the square of the dielectric breakdown strength and the dielectric constant [10]. However, for common homogeneous polymer dielectrics, there is an inherent contradiction that the breakdown strength and dielectric constant [7, 11, 12] or high-temperature performance [13] are difficult to improve simultaneously. For example, biaxially oriented polypropylene (BOPP), one of the most successful energy storage polymer dielectrics currently commercialized, has a very high breakdown strength (approximately 700 MV m^− 1^). However, its low dielectric constant (approximately 2.2) has become a key factor restricting improvement of its energy storage density, and its long-term operating temperature needs to be lower than 85 °C; thus, an additional cooling system needs to be installed for cooling [13, 14]. In recent years, although good results have been achieved through nanomaterial compounding [1, 15–19] and new molecular design [13], using these methods in industry remains difficult due to the material cost and complex preparation process. The changes in the electrical strength are related to the material charge properties, which are directly related to carrier trapping [20, 21]. However, the current research lacks microscopic and direct observations of polymer carrier traps, which leads to the inability to well explain the microscopic mechanism of the electric intensity changes.

Therefore, an orientation-distributed inorganic–organic sandwich-like coating that can be applied to the surface of polymer dielectrics is obtained by simple self-assembly of boron nitride nanosheets (BNNSs). We applied the coating with wide bandgap to different kinds of polymers and found that the breakdown strength of the polymer dielectrics significantly increased after the coating was applied. We further observed the charge characteristics and trap characteristics of the polymers at the nanoscale based on Kelvin probe force microscopy (KPFM) and found that the coating can reduce the surface trap energy level and promote evacuation of injected charges to the surrounding area. This explains why the coating can improve the flashover voltage. The dielectric spectra at different temperatures and different frequencies show that the dielectric constant and thermal stability of the polymer dielectrics are improved after coating. Through density functional theory (DFT) calculations, the experimental results are validated. Decoupling regulation of the breakdown strength, dielectric constant and thermal stability is achieved. The method of researching the microscopic carrier trap characteristics proposed in this paper provides guidance for in-depth study of the electrical strength of polymer dielectrics, and the proposed coating can also improve the performance of polymer dielectrics.

## Results and discussion

### Preparation and characterization

**Fig. 1:**
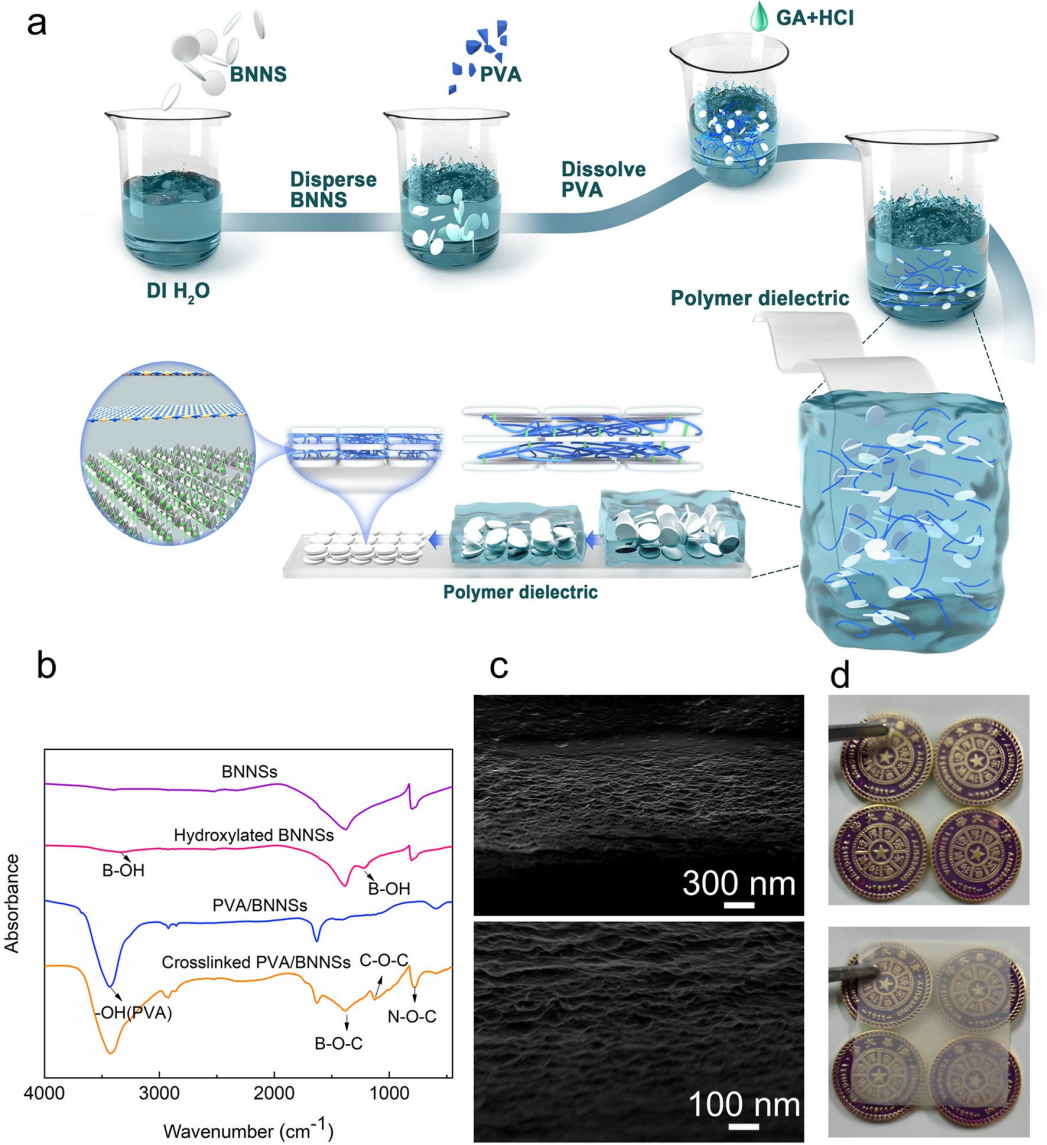
Sample preparation process and characterization. **a** Schematic diagram of the coating preparation process. **b** FTIR spectra of BNNSs before and after hydroxylation and before and after the PVA/BNNS cross-linking reaction. **c** SEM images of the cross section of the coating. **d** Photographs of the BOPP film before and after coating (top, uncoated; bottom, coated)

A schematic diagram of the coating preparation method is shown in Fig. 1a. Based on BNNSs (all BNNSs discussed in this paper are hydroxylated BNNSs, see the Experimental Methods section) and polyvinyl alcohol (PVA), the addition of the cross-linking agents glutaraldehyde (GA) and hydrochloric acid (HCl) for catalysis will induce the self-assemble of a film on a surface to form an oriented inorganic–organic coating similar to sewing clothes (the detailed preparation process of the coating is shown in the experimental methods section, and the chemical reaction schematic diagram is shown in Additional file 1: Fig. S1). To explore the generalizability of this coating for improving the performance of polymer dielectrics, we applied the coating to four common polymer dielectric surfaces in industry: BOPP, polyimide (PI), low density polyethylene (LDPE) and polytetrafluoroethylene (PTFE). The thicknesses of the four polymer dielectrics were all 28 μm, and the coating thickness was controlled at 1 μm. The Fourier transform infrared (FTIR) spectra shown in Fig. 1b indicate that new absorption peaks appeared at 1220 cm^−1^ and 3336 cm^−1^ after hydroxylation of BNNSs. The characteristic absorption peaks correspond to the in-plane and tensile bending of the B–OH bond. –OH was proven to be successfully grafted onto the surface of BNNSs after hydroxylation treatment, which was also confirmed by thermogravimetric analysis (TGA) (Additional file 1: Figure S2). After the cross-linking reaction between PVA and BNNSs, new peaks appeared at 890 cm^−1^, 1187 cm^−1^ and 1382 cm^−1^, implying the formation of N–O–C, C–O–C and B–O–C bonds, which also confirmed that the cross-linking reaction could occur during the self-assembly process. The scanning electron microscopy (SEM) image shown in Fig. 1c visually presents the cross-sectional structure of the coating. Additional file 1: Fig. S3 shows the SEM image of the polymer surface after coating, and the coating is applied to the polymer surface. The coating showed a milky white translucent feature after being coated on the BOPP film (see Fig. 1d), and photos of the coatings on other dielectric films are shown in Additional file 1: Fig. S4.

### Electric strength test

**Fig. 2:**
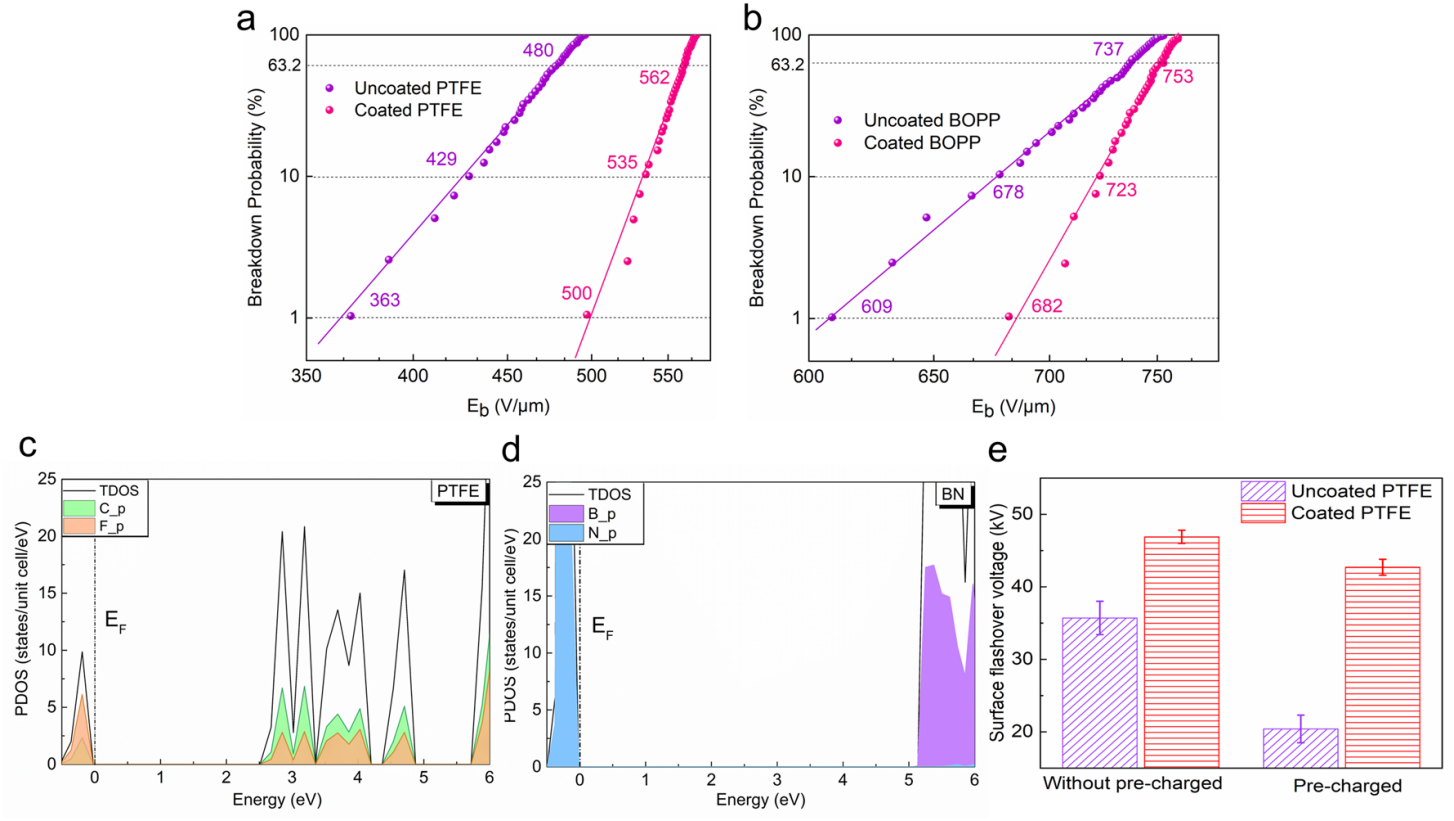
Breakdown strength test, DFT calculation and flashover voltage. Weibull distribution of breakdown strengths of uncoated and coated **a** PTFE, **b** BOPP. Density of state calculated by the HSE + SOC method for **c** the (001) surface of PTFE. **d** the (001) surface of BN. The Fermi level is set to 0. **e** Flashover voltage of coated and uncoated PTFE

As shown in Fig. 2a–d, we used Weibull statistics to analyze the breakdown strengths (E_b_) of different kinds of polymer dielectric films before and after coating, and we determined the values for which the cumulative failure probability was 1% (the maximum electric field for practical safe use), 10–63.2% (commonly used to compare the breakdown strength of various samples). The slope β is the shape coefficient, which represents the dispersion of the breakdown strength, and the larger β is, the smaller the dispersion. We can see that there is a significant improvement in both the breakdown strength and shape factor after applying the coating. Take PTFE, known as the “King of Plastics”, which is widely used in the electrical industry, aerospace, and electronic devices as an insulating layer for power and signal lines, as an example (Fig. 2a). The E_b_ value for the 1% breakdown probability is increased from 363 V/μm to 500 V/μm, an increase of 37.7%. The E_b_ value for the 63.2% breakdown probability is increased from 480 V/μm to 562 V/μm, an increase of 17%. In addition, the β value is also increased from 20 to 45, an increase of 125%, indicating that the dispersion of E_b_ is reduced, which effectively improves the reliability of the dielectric. (For detailed Weibull analysis data, see Additional file 1: Table S1) In addition to polymer dielectrics with low breakdown strength, the coating improves the breakdown strength even for dielectrics such as BOPP that are widely used in energy storage capacitors due to their high breakdown strength. As shown in Fig. 2b, the E_b_ value for the 1% breakdown probability increases from 609 V/μm to 682 V/μm, an increase of 11.9%. The E_b_ value for the 63.2% breakdown probability increases from 737 V/μm to 753 V/μm, an increase of 2.1%. The β value also increases from 27 to 55, an increase of 103%.

To investigate the reason for the improved breakdown strength, we coated the dielectric polymer only with the organic layers (PVA and GA) in a sandwich structure. No significant improvement in breakdown strength was observed (see Additional file 1: Fig. S5–S8). The density of state was calculated with the HSE + SOC DFT method, and the results show that the band gaps of PTFE and BN were 2.5 eV and 5.1 eV, respectively (as shown in the Fig. 2). The results indicate that the breakdown field strength of PTFE with BN was enhanced, because the coating was the first to make contact with the breakdown field strength, and a larger band gap led to a greater breakdown field strength.^13^ This result also shows that, based on the wide bandgap, the BNNS coating can effectively improve the breakdown strength of the dielectric polymer. In addition, the ability of the coating to effectively improve the electrical strength of the dielectric polymer when the thickness is reduced is encouraging (see Additional file 1: Figs. S9–S12). In this study, we also prepared 600 nm thick coatings for application to different kinds of dielectric surfaces. Although the E_b_ values were slightly lower than those for the 1 μm coating, they remained much higher than those of the uncoated dielectrics, and the β value was basically the same as that of the dielectric material coated with the 1 μm coating.

The flashover voltage is another important parameter in insulation systems. Since the interface between the dielectric and other media in an insulation system has a lower breakdown voltage than when they exist alone, increasing the flashover voltage can effectively improve the safety and reliability of the device [22–24]. We performed flashover voltage experiments with PTFE, commonly used for insulation, as an example, and the results are shown in Fig. 2e. When the flashover experiment is directly performed without charge accumulation on the surface, the flashover voltage of PTFE is 35.7 kV. When the coating is applied, the flashover voltage is increased to 46.9 kV, an increase of 31.3%. This is more prominent than the increase with some of the current best methods [25–28]. However, polymer dielectrics will accumulate a large amount of charge on the surface during actual use [29, 30] causing distortion of the surface electric field and resulting in a significant drop in flashover voltage [31, 32]. Therefore, when the surface is precharged, the flashover voltage of PTFE drops to 20.4 kV, and the drop is as high as 57%. However, when the coating is applied, the flashover voltage only drops to 42.7 kV, still maintaining an outstanding level of insulation.

### Microscopic charge and trap parameter testing

**Fig. 3:**
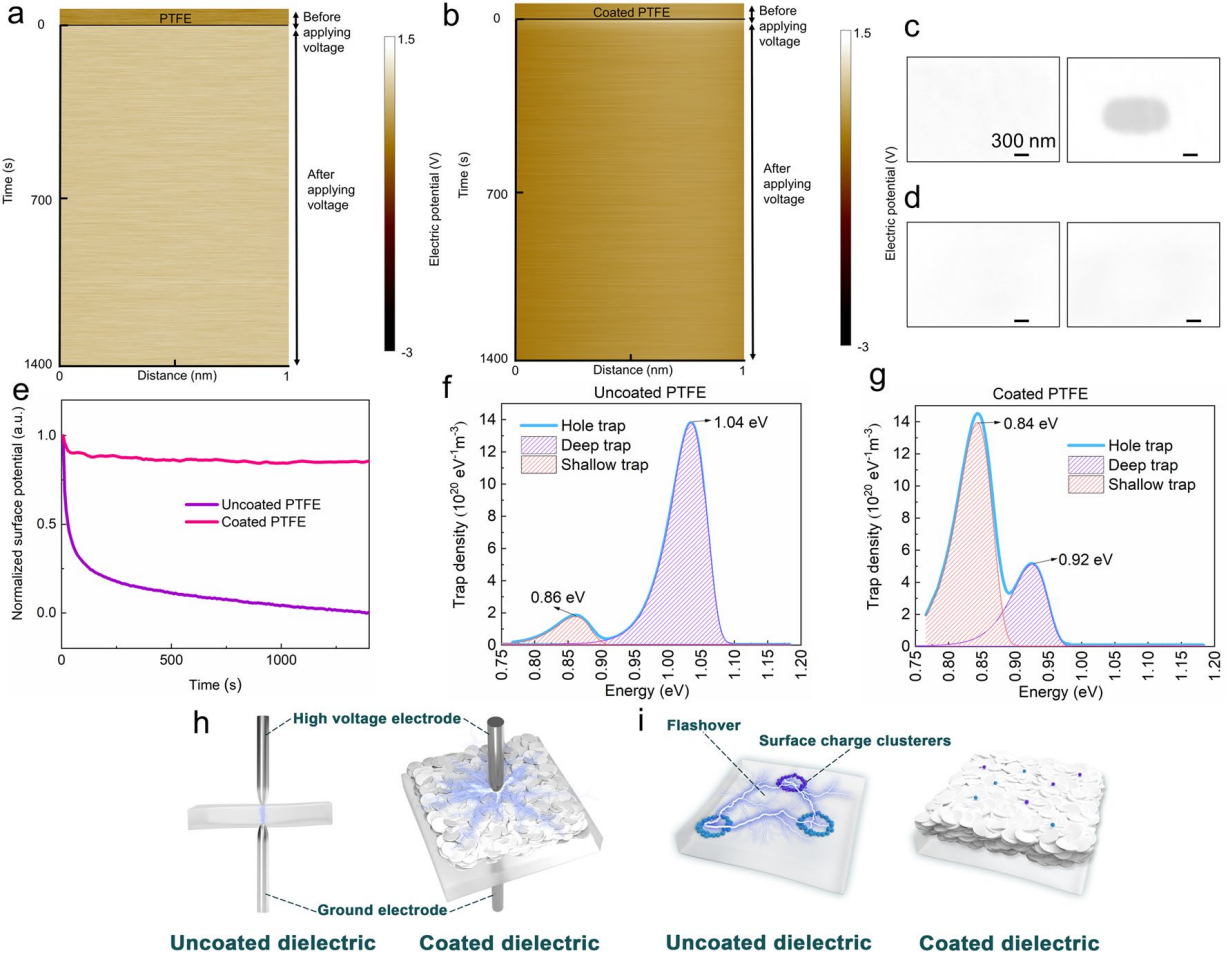
Charge characteristics of nanodomains, trap parameters, and schematic diagram of the coating to enhance the electrical strength of polymer dielectrics. **a** Surface potential dissipation map of PTFE based on KPFM. **b** Surface potential dissipation map of coated PTFE based on KPFM. **c** Images of PTFE before (left) and after (right) charging by SEM. **d** Images of coated PTFE before (left) and after (right) charging by SEM. **e** Normalized surface potential dissipation curves of PTFE before and after coating. **f** PTFE trap parameters, **g** Coated PTFE trap parameters. **h** Schematic diagram of the mechanism by which the coating enhances the breakdown strength. **i** Schematic diagram of the coating to enhance the flashover voltage

The electrical strength of polymer dielectrics is related to the charge transport properties, which are determined by polymer carrier trapping. Based on KPFM, we investigated the charge behavior in the nanodomains before and after polymer coating. Figure 3a shows that the potential is very stable when the PTFE surface is charged, which indicates that the charge is hardly dissipated after accumulating on the surface of the material. When the coating is applied, as shown in Fig. 3b, the accumulated charge rapidly dissipates. Other types of polymer dielectrics show very similar rapid dissipation of surface charge after being coated with this coating (see Additional file 1: Figs. S13–S16). This result was verified by SEM. Figure 3c shows the PTFE sample before coating. The electrons in SEM form a charge spot with high electron density after hitting the PTFE surface. However, after applying the coating, as shown in Fig. 3d, there is almost no electron deposition. Based on the experimental results in Fig. 3a, b, we can obtain the normalized dissipation curve of the sample surface potential shown in Fig. 3e. According to this dissipation curve, the trap parameters of the sample can be obtained [20, 33]. As shown in Fig. 3f, g, the deep trap energy level of the surface decreases from 1.04 eV to 0.92 eV after PTFE is coated. The deep trap density drops from 14 × 10^20^ eV^−1^ m^−3^ to 5 × 10^20^ eV^−1^ m^−3^. The shallow trap density exhibits an opposite increasing trend. The above results show that the coating is equivalent to introducing a large number of shallow traps on the surface of the material, which promotes dissipation of charges along the surface. Since BNNSs have a wide bandgap, the oriented coating can maintain a high dielectric strength in the normal direction and inhibit charge injection. The inorganic–organic sandwich-like structure enables facilitation of charge dissipation in the tangential direction. In this way, forming electrical branches under the action of a strong electric field and causing breakdown is difficult for the polymer dielectric (Fig. 3h). Distortion of the electric field and induction of flashover due to the accumulation of a large amount of surface charges during use is also difficult (Fig. 3i).

### Dielectric performance test

**Fig. 4:**
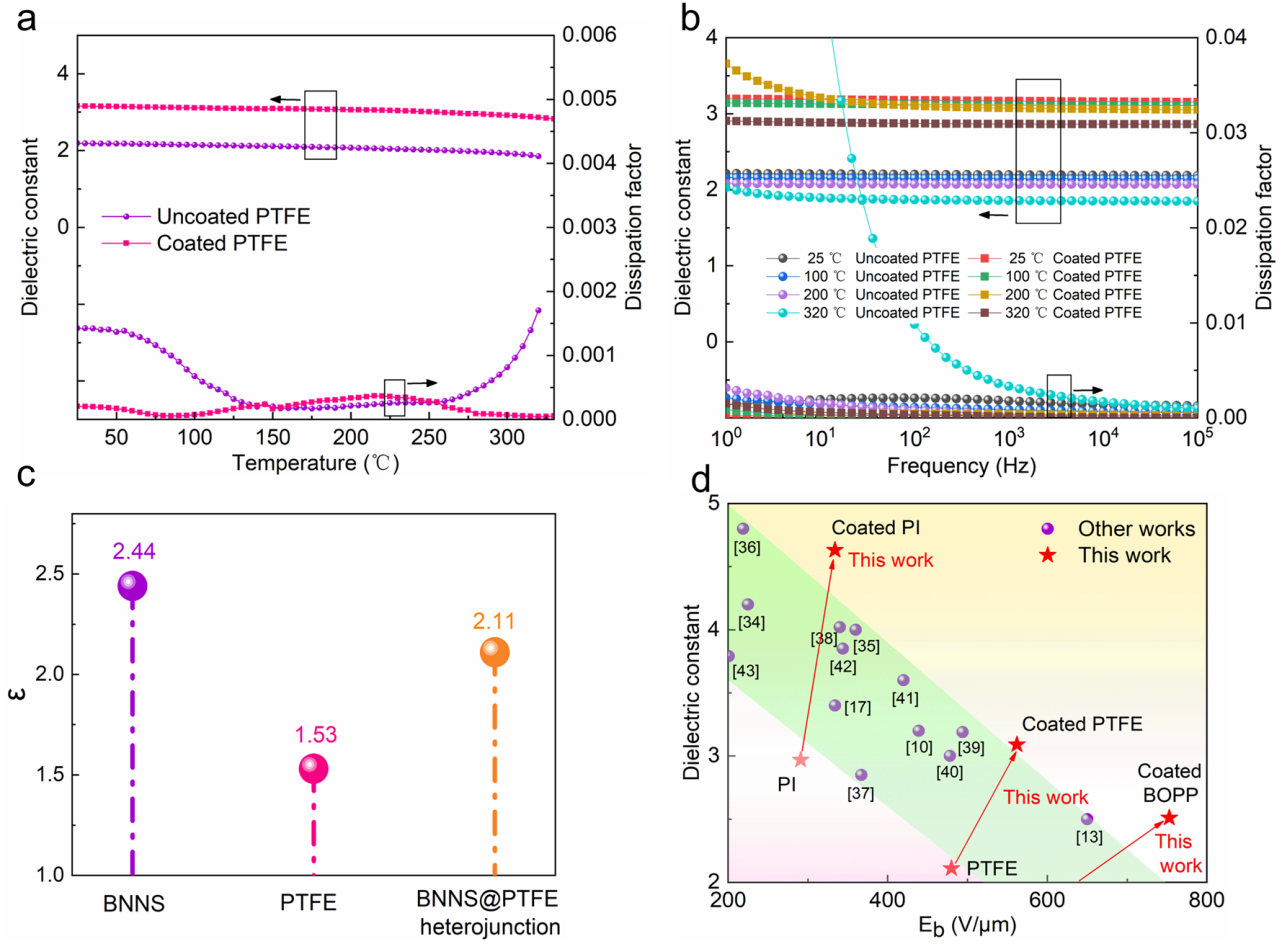
Dielectric performance test and DFT calculation. **a** Changes in the K and DF values of PTFE with temperature (frequency of 10.^4^ Hz) before and after coating. **b** Changes in the K and DF values of PTFE with frequency at different temperatures before and after coating. **c** The average static dielectric constant of the (001) surface of BN, the (001) surface of PTFE, and the BNNS@PTFE heterojunction calculated by the DPFT method. **d** The breakdown strength and dielectric constant of polymer dielectrics analyzed in the present study and reported in the literature at high temperature (150 °C)

We evaluated the change in the dielectric constant (K) and dissipation factor (DF) of different polymer dielectrics before and after coating at different temperatures and frequencies. Figure 4a shows that at 10^4^ Hz (a common power regulation frequency^1^), the K value of PTFE is significantly increased after coating. At room temperature, it improved from 2.19 to 3.16, a 44% increase, which is quite impressive. To investigate the reason for the improved K value, we coated the dielectric polymer only with the organic layers (PVA and GA) in the sandwich structure. No significant improvement in K value was observed (see Additional file 1: Figs. S17–S20). We verified the experimental results by means of DFT calculation. The dielectric constant with the method of density functional perturbation theory (DFPT) was calculated as shown in Fig. 4c and Additional file 1: Table S2 of the Supporting Information (Slab prototypes of the (001) surface of BN, the (001) surface of PTFE, and BNNS@ PTFE heterojunction see Additional file 1: Fig. S21). The ε of (001) surface of BN, pure (001) surface of PTFE, and BNNS@ PTFE heterojunction were 2.44, 1.53, 2.11. Such results suggest that the static dielectric constant of the system tended to increase after the introduction of the BN layer coating, which was also consistent with the experimental phenomena. The increase in the K value was also observed after coating other types of dielectrics (Additional file 1: Figs. S22–S24). In addition, with changing temperature, the K and DF values of PTFE are also more stable after coating; that is, it shows more stable dielectric properties. This is due to the good heat dissipation performance of BNNSs. For example, at 325 °C, the K value of the coated PTFE changes by only 9.92% relative to that at room temperature. This value is much lower than the change in the value for the uncoated PTFE of 130.28%, indicating that it is completely unusable at this point (Additional file 1: Fig. S25). For other types of polymer dielectrics, the K and DF values also show better temperature stability after coating, and the maximum operating temperature is also improved (Additional file 1: Figs. S22–S26). Similar to the temperature characteristics, the result that the coated PTFE exhibits very stable K and DF values over a wide frequency range of 10^0^ Hz–10^5^ Hz, whether at room temperature or high temperature, is also outstanding (Fig. 4b, Additional file 1: Fig. S27). The K value is always higher than that of the uncoated PTFE sample under the same conditions. The frequency stability of other polymer dielectrics after coating was also verified (Additional file 1: Figs. S28–S30). In addition, when the thickness of the coating is decreased to 600 nm, the polymer dielectric still achieves very good high-temperature dielectric properties, but the dielectric constant is slightly lower than that for the 1 μm coating, although it is still significantly higher than that of the uncoated sample (Additional file 1: Figs. S31–S34).

Figure 4d shows the values of the breakdown strength and dielectric constant values for the high-temperature polymer dielectrics reported in this paper and in the published literature at high temperature (150 °C) [10, 13, 17, 34–43]. The dielectric constant and breakdown field strength of the most widely used polymer dielectrics are significantly improved at high temperatures. For example, the BOPP film, which is widely used for energy storage, can no longer be used normally at 150 °C. In the present study, BOPP still maintains very good performance at 150 °C. Other examples are a polyimide (PI) film and polytetrafluoroethylene (PTFE) film, which are currently widely used in high-temperature environments and insulation. Although they can still be used normally at 150 °C, after the modification described in this paper, their breakdown field strength at 150 °C is increased by 14.7–17.1%, and the dielectric constant is increased by 55.8% and 46.4%, respectively. These improvements are very impressive.

## Conclusions

In conclusion, in this study, an oriented inorganic–organic coating with a wide bandgap that can be coated on different kinds of polymer dielectrics is prepared by facile self-assembly of BNNSs. After the coating is applied, the breakdown strength and flashover voltage of the polymer dielectrics are significantly improved. Based on KPFM, the charge transfer characteristics of nanodomains are studied, and after coating, a large number of shallow traps are introduced onto the surface of the material, which promote dissipation of injected charges. This also explains the increase in electrical strength after applying the coating. In addition, the dielectric property tests at different temperatures and frequencies show that the dielectric constant of the polymer dielectrics is significantly improved after coating, and good high-temperature dielectric properties are obtained. The coated dielectrics can maintain stable K and DF values at high temperatures and over wide frequencies. Density functional theory (DFT) calculations validate the experimental results and explain the reason for the increase in breakdown strength and dielectric constant after coating. Whether the polymer dielectric is used as an insulating system or an energy storage material, the coating can effectively improve its application performance.

## Methods

### Materials

BNNSs (98% purity, 100 nm–400 nm flake diameter) were purchased from XFNANO, China. PVA (molecular weight (MW): 1176000) and GA (50 wt % solution, MW: 100.12) were purchased from Energy Chemical, China. HCl (36–38 wt % solution) was purchased from Beijing Chemical Factory Company, China. The polymer dielectrics were all provided by the School of Materials, Tsinghua University, with a thickness of 28 μm.

### Coating preparation

The BNNSs were first hydroxylated. One hundred grams of NaOH was dissolved in 500 ml of deionized (DI) water. Then, 5 g of BNNSs was dispersed in a NaOH solution at 120 °C for 48 h with a dispersant at a speed of 300 r/min to hydroxylate them. The mixture was rinsed with DI water and filtered to pH 7. Finally, the product was dried at 80 °C for 12 h to remove residual moisture. After hydroxylation of BNNSs, the samples before and after hydroxylation were characterized by FTIR spectroscopy and TGA (Fig. 1b, Additional file 1: Fig. S2). The results proved that -OH was successfully grafted to the surface of BNNSs after hydroxylation. These characterization results are also in good agreement with previous findings on the hydroxylation of BNNSs [44–46]. The fabrication process of the coating is shown in Fig. 1a. The hydroxylated BNNSs were poured into DI water and dispersed with a high-speed disperser at 3000 rpm for 10 min to obtain a suspension with a mass fraction of 1%. PVA particles with the same mass as BNNSs were then poured into DI water at 95 °C and dispersed with a high-speed disperser at a speed of 3000 rpm for 10 min to obtain a solution with a mass fraction of 1%. After cooling the PVA solution to room temperature, it was poured into the BNNS suspension and dispersed at 3000 rpm for 20 min, followed by ultrasonic dispersion for 10 min (40 kHz, 360 W). Then, GA and HCl at 0.1 times and 0.3 times the mass of PVA, respectively, were added, dispersed at 1000 rpm for 5 min, and then dispersed by ultrasonication for 5 min. Since polymer dielectric surfaces tend to be hydrophobic, they needed to be treated with a dielectric barrier discharge (DBD) plasma to increase their surface hydrophilicity before applying the coating. Argon was used as the excitation gas, the gas flow was 9 l/min, the polymer dielectric was placed 3 cm from the plasma outlet, and each sample was processed for 60 s. Then, the polymer dielectric film was vertically immersed in the above solution for 20 s, vertically removed, placed in a drying oven at 60 °C for 30 min, removed, dipped and dried. In this study, we repeated the above dipping-drying steps 4 or 2 times to obtain a coating with a thickness of approximately 1 μm or 600 nm.

### Material characterization

Coating sections were observed with SEM (GeminiSEM 500, Zeiss). FTIR spectra (Nicolet 6700) were measured from 650 to 4000 cm^−1^ at room temperature. Under the protection of nitrogen, the measurement temperature of TGA was from room temperature to 800 °C, and the heating rate was 20 °C/min.

### Electric strength test

Breakdown strength test. The contact area of the high-voltage electrode and the ground electrode with the polymer dielectric was 15 mm × 15 mm. The high-voltage electrode was connected to a DC high-voltage power supply with a boost rate of 200 V/s until breakdown. The voltage value at the instant of breakdown was the current breakdown voltage, and the breakdown strength was calculated according to the thickness of the sample. The same sample was tested fifty times, and the breakdown strength data were analyzed by the two-parameter Weibull distribution. Its probability density function can be expressed as:

1 $$P\left( E \right) = 1 - \exp \left[ { - \left( {\frac{E}{{E_{0} }}} \right)^{\beta } } \right]$$

Here, P(E) is the cumulative probability value of the breakdown of the sample; E is the actual breakdown strength; β is the shape coefficient, representing the dispersion of the breakdown field strength, in which the larger β is, the smaller the dispersion of the breakdown strength of the sample; and E_0_ is the scale parameter of the Weibull distribution, representing the corresponding breakdown strength when P is 63.2%. E_0_ is usually taken as the average breakdown strength of the test sample.

Flashover voltage test. Two hemispherical copper electrodes with a diameter of 30 mm were used as the high voltage electrode and the ground electrode. The two electrodes were passed through a pressing device to achieve close contact between the sample and the electrodes. The distance between the two electrodes was 20 mm. Each type of sample was tested thirty times at a boost rate of 200 V/s until flashover occurred. For the precharged test group, the needle-plate electrode was used for corona discharge, the needle electrode was located 2 mm above the sample, and a voltage of − 3 kV was applied to precharge the surface of the sample. Then, a positive voltage was applied through the above steps to perform a flashover test.

### KPFM test

The KPFM test was based on a Bruker Scientific Dimension FastScan model multifunctional scanning probe microscope. The sample was fixed on the sample stage with conductive tape, and the edge of the sample and the sample stage were connected with conductive copper tape to provide a channel for charge dissipation. The KPFM test used a PFQNE-AL-type probe, the main scan used peak force tapping, the probe lift height was the default value of 114.5 nm, the scan rate was 0.5 Hz, the sample number was 256, the scan size was 1 nm, and the aspect ratio was 70. One frame took approximately 7 s to scan. The probe was first tested without bias for 10 frames (70 s). Then, a + 3 V bias was applied to the probe for 10 min to inject charges onto the surface of the sample, and the sample number was changed to 2048 when the bias was applied. Since we only need the potential values before and after the voltage is applied, to speed up the scan rate when biased, the potential channel was closed when biased. After the voltage application was completed, the bias voltage was removed, the sample number was changed back to 256, the potential signal channel was opened, and 200 frames were scanned (1400 s) to obtain the potential dissipation curve. The dissipation curve was normalized, and then, the trap parameters were calculated by the isothermal surface potential decay (ISPD) method according to the measured normalized potential dissipation curve [20, 33, 47–49].

### Dielectric performance test

The dielectric performance test was based on a broadband dielectric spectrometer (Novocontrol Concept 40). The sample thickness was precisely measured and gold was sprayed before each measurement. The measurement frequency range was 10^0^–10^5^ Hz. The temperature was measured every 5 °C starting from 25 °C, and the temperature was raised until the dielectric spectrum showed serious abnormality or the sample was damaged.

### DFT calculations

The density functional theory calculations were implemented in the Vienna ab initio Simulation Pack (VASP) code [50]. The projector-augmented wave (PAW) approach was used to describe electron–ion interactions [51]. The generalized gradient approximation of the Perdew-Burke-Ernzerhof (PBE) functional and the Heyd-Scuseria-Ernzerhof (HSE06) hybridization function were employed for geometry optimizations and self-consistent static calculations, respectively [52–56]. Spin–orbit coupling (SOC) was considered. The kinetic energy cutoff was set to 520 eV. A conjugate gradient method was applied for geometry optimizations, with a Gaussian smearing width of 0.05 eV. The total energy criterion in the electronic self-consistency loop and the force criteria in the ionic relaxation loop was set to 10^–5^ eV and 0.02 eV Å^−1^, respectively.

In the calculations of density of state, supercells of BN’s (001) surface with 180 atoms, and PTFE’s (001) surface with 240 atoms were used, in combination with a Γ-centered 2 × 2 × 1 k-mesh. In the calculations of the dielectric constant, the model of PTFE’s (001) surface and BNNS@ PTFE heterojunction were used with the DFPT calculation method. Slab prototypes were constructed beyond 4 atomic layers, above which a vacuum space of 15 Å in the perpendicular direction was added to prevent interactions between neighboring slabs, as shown in Additional file 1: Fig. S21.

## Supplementary Information

**Additional file 1: Table S1.** Breakdown Strength Weibull Analysis Detailed Numerical Values. **Table S2.** Static dielectric constants ε_*II*_, ε_*JJ*_, and ε_*KK*_ in three principal directions and the average static dielectric constant ε_av_ of the (001) surface of BNNS, the (001) surface of PTFE, and the BNNS@PTFE heterojunction. **Figure S1.** Schematic diagram of the chemical reaction principle of the self-assembly of BNNSs. **Figure S2.** Thermogravimetric analysis of BNNSs before and after hydroxylation. **Figure S4.** Photographs of polymer dielectric films before (top) and after (bottom) coating. (a) PI, (b) LDPE, (c) PTFE. **Figure S5.** Weibull distribution of the breakdown strengths of uncoated and PVA-coated PTFE. **Figure S6.** Weibull distribution of the breakdown strengths of uncoated and PVA-coated BOPP. **Figure S7.** Weibull distribution of the breakdown strengths of uncoated and PVA-coated PI. **Figure S8.** Weibull distribution of the breakdown strengths of uncoated and PVA-coated LDPE. **Figure S9.** Weibull distribution of the breakdown strengths of uncoated and coated PTFE with different thicknesses. **Figure S10.** Weibull distribution of the breakdown strengths of uncoated and coated BOPP with different thicknesses. **Figure S11.** Weibull distribution of the breakdown strengths of uncoated and coated PI with different thicknesses. **Figure S12.** Weibull distribution of the breakdown strengths of uncoated and coated LDPE with different thicknesses. **Figure S13.** KPFM-based surface potential dissipation diagram of coated BOPP. **Figure S14.** KPFM-based surface potential dissipation diagram of coated PI. **Figure S15.** KPFM-based surface potential dissipation diagram of coated LDPE. **Figure S16.** Normalized surface potential dissipation curves based on KPFM for different kinds of polymer dielectrics after coating. **Figure S17.** K and DF values of uncoated and PVA-coated PTFE. **Figure S18.** K and DF values of uncoated and PVA-coated BOPP. **Figure S19.** K and DF values of uncoated and PVA-coated PI. **Figure S20.** K and DF values of uncoated and PVA-coated LDPE. **Figure S21.** Slab prototypes of (a) the (001) surface of BNNS, (b) the (001) surface of PTFE, and (c) BNNS@ PTFE heterojunction. The atoms are represented by spheres: N (blue), B (yellow), C (green), and F (white). **Figure S22.** Changes in the K and DF values of BOPP with temperature (frequency of 10^4^ Hz) before and after coating. **Figure S23.** Changes in the K and DF values of PI with temperature (frequency of 10^4^ Hz) before and after coating. **Figure S24.** Changes in the K and DF values of LDPE with temperature (frequency of 10^4^ Hz) before and after coating. **Figure S25.** Percent change in the K values of PI and PTFE before and after coating at different temperatures relative to those at 25 °C (frequency of 10^4^ Hz). **Figure S26.** Percent change in the K values of BOPP and LDPE before and after coating at different temperatures relative to those at 25 °C (frequency of 10^4^ Hz). **Figure S27.** Percent change in the K value of polymer dielectrics before and after coating at extreme temperatures relative to 25 °C at different frequencies. **Figure S28.** Changes in the K and DF values of BOPP with frequency at different temperatures before and after coating. **Figure S29.** Changes in the K and DF values of PI with frequency at different temperatures before and after coating. **Figure S30.** Changes in the K and DF values of LDPE with frequency at different temperatures before and after coating. **Figure S31.** K and DF values of PTFE coated with films of different thicknesses as a function of temperature (frequency of 10^4^ Hz). **Figure S32.** K and DF values of BOPP coated with films of different thicknesses as a function of temperature (frequency of 10^4^ Hz). **Figure S33.** K and DF values of PI coated with films of different thicknesses as a function of temperature (frequency of 10^4^ Hz). **Figure S34.** K and DF values of LDPE coated with films of different thicknesses as a function of temperature (frequency of 10^4^ Hz).

## Data Availability

The data that support the findings of this study are available from the authors on reasonable request.
