# Supplementary material for: Self-assembled wide bandgap nanocoatings enabled outstanding dielectric characteristics in the sandwich-like structure polymer composites
Source: Nano Converg. 2022 Dec 9;9:55. doi: 10.1186/s40580-022-00346-2 (PMC9733754; doi:10.1186/s40580-022-00346-2)
Supplement: Supplementary file 1 — Additional file 1: Table S1. Breakdown Strength Weibull Analysis Detailed Numerical Values. Table S2. Static dielectric constants εII, εJJ, and εKK in three principal directions and the average static dielectric constant εav of the (001) surface of BNNS, the (001) surface of PTFE, and the BNNS@PTFE heterojunction. Figure S1. Schematic diagram of the chemical reaction principle of the self-assembly of BNNSs. Figure S2. Thermogravimetric analysis of BNNSs before and after hydroxylation. Figure S4. Photographs of polymer dielectric films before (top) and after (bottom) coating. (a) PI, (b) LDPE, (c) PTFE. Figure S5. Weibull distribution of the breakdown strengths of uncoated and PVA-coated PTFE. Figure S6. Weibull distribution of the breakdown strengths of uncoated and PVA-coated BOPP. Figure S7. Weibull distribution of the breakdown strengths of uncoated and PVA-coated PI. Figure S8. Weibull distribution of the breakdown strengths of uncoated and PVA-coated LDPE. Figure S9. Weibull distribution of the breakdown strengths of uncoated and coated PTFE with different thicknesses. Figure S10. Weibull distribution of the breakdown strengths of uncoated and coated BOPP with different thicknesses. Figure S11. Weibull distribution of the breakdown strengths of uncoated and coated PI with different thicknesses. Figure S12. Weibull distribution of the breakdown strengths of uncoated and coated LDPE with different thicknesses. Figure S13. KPFM-based surface potential dissipation diagram of coated BOPP. Figure S14. KPFM-based surface potential dissipation diagram of coated PI. Figure S15. KPFM-based surface potential dissipation diagram of coated LDPE. Figure S16. Normalized surface potential dissipation curves based on KPFM for different kinds of polymer dielectrics after coating. Figure S17. K and DF values of uncoated and PVA-coated PTFE. Figure S18. K and DF values of uncoated and PVA-coated BOPP. Figure S19. K and DF values of uncoated and PVA-coated PI. Figure S20. K and DF [file 40580_2022_346_MOESM1_ESM.docx]

Additonal file

**Self-assembled wide bandgap nanocoatings enabled outstanding dielectric characteristics in the sandwich-like structure polymer composites**

Tian-Yu Wang^1, †^, Xiao-Fen Li^2, †^, Shu-Ming Liu^1^, Bai-Xin Liu^2^, Xi-Dong Liang^1^, Shunning Li^3^, Gui-Xin Zhang^1,^ ^*^, Jian-Bo Liu^2, *^, Zhi-Min Dang^1, *^

^†^These authors contributed equally: Tian-Yu Wang, Xiao-Fen Li

^1^State Key Laboratory of Control and Simulation of Power System and Generation Equipment, Department of Electrical Engineering, Tsinghua University, Beijing, 100084 China

^2^ Key Laboratory of Advanced Materials (MOE), School of Materials Science and Engineering, Tsinghua University, Beijing 100084, China

^3^ School of Advanced Materials, Peking University, Shenzhen Graduate School, Shenzhen 518055, China

*Email: [guixin@mail.tsinghua.edu.cn](mailto:guixin@mail.tsinghua.edu.cn); [jbliu@mail.tsinghua.edu.cn](mailto:jbliu@mail.tsinghua.edu.cn); [dangzm@tsinghua.edu.cn](mailto:dangzm@tsinghua.edu.cn)

**
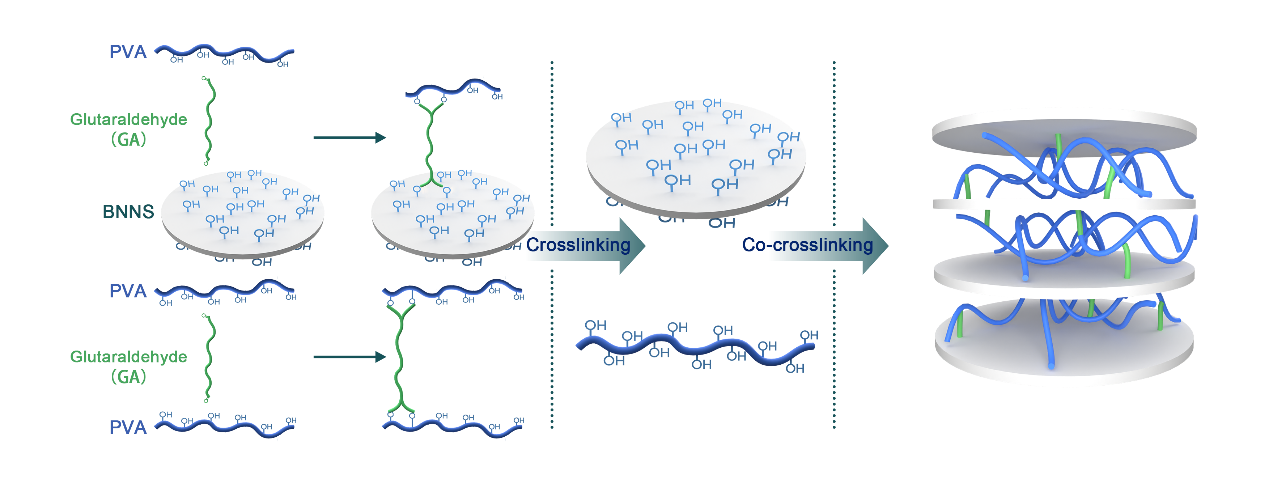
**

**Figure S1.** Schematic diagram of the chemical reaction principle of the self-assembly of BNNSs.

**
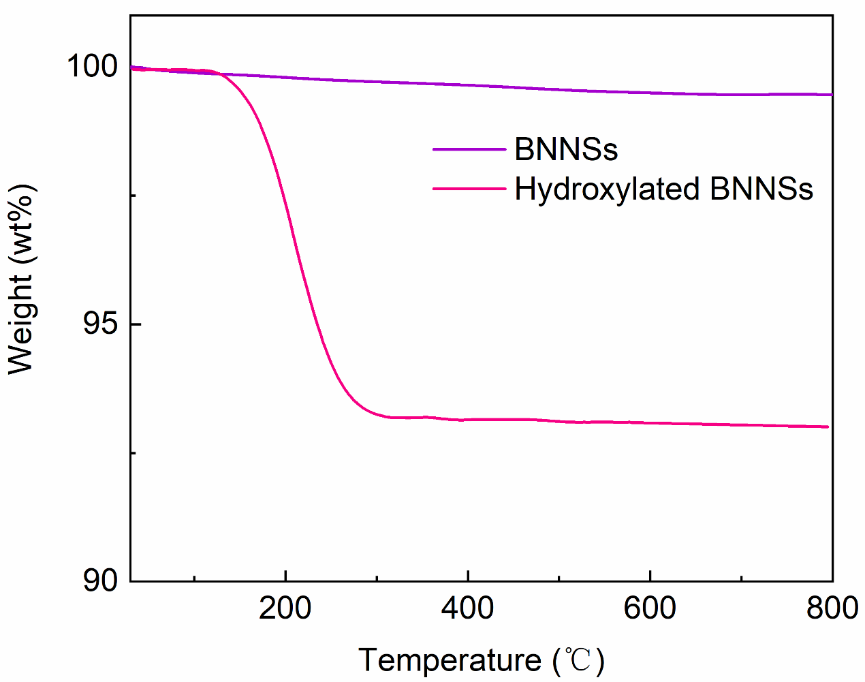
**

**Figure S2.** Thermogravimetric analysis of BNNSs before and after hydroxylation.

**
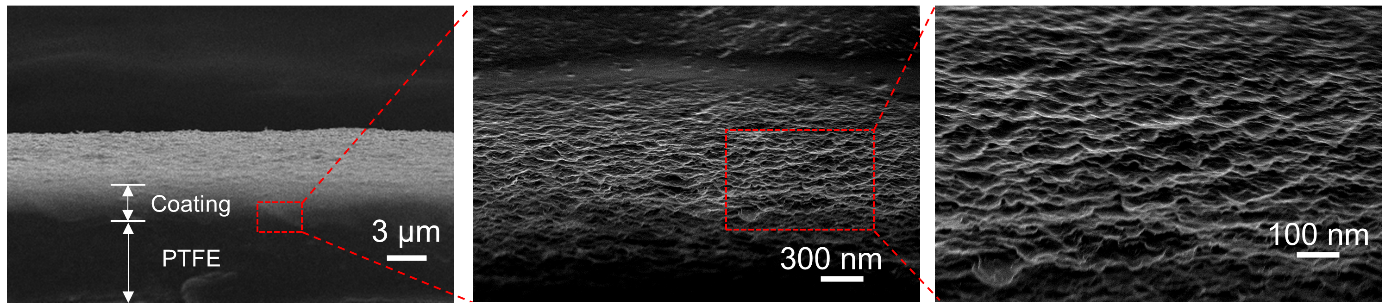
**

**Figure S3.** SEM images of the cross section of the polymer surface coating.

**
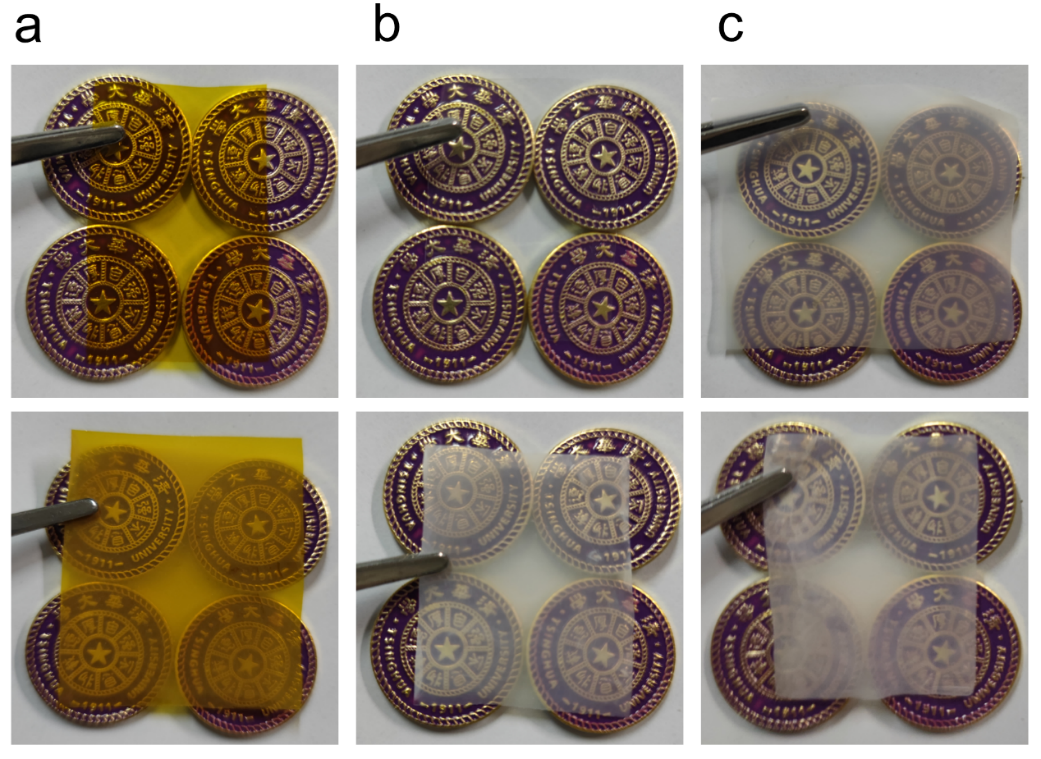
**

**Figure S4.** Photographs of polymer dielectric films before (top) and after (bottom) coating. (a) PI, (b) LDPE, (c) PTFE.


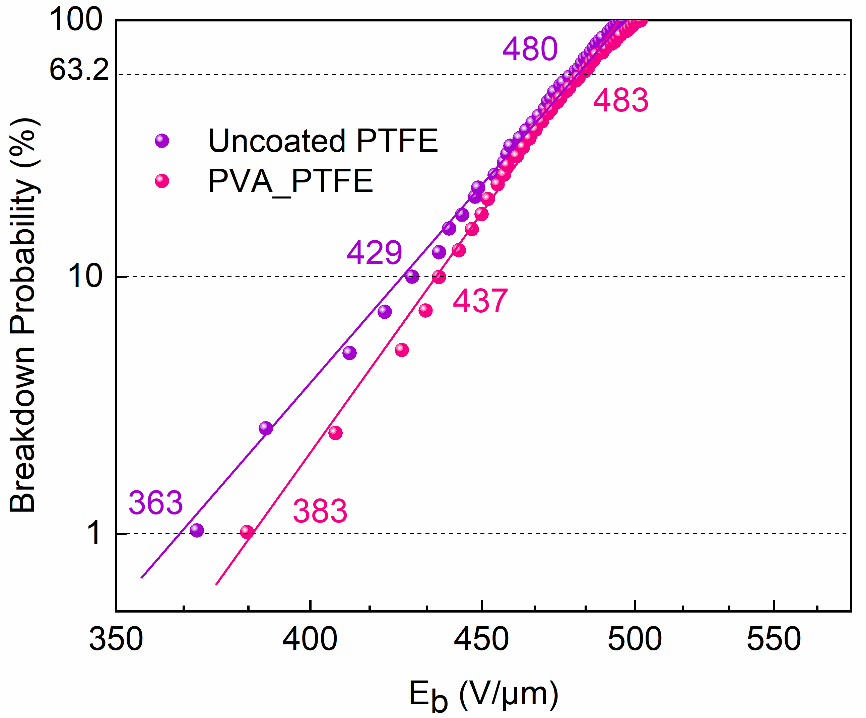


**Figure S5.** Weibull distribution of the breakdown strengths of uncoated and PVA-coated PTFE.

**
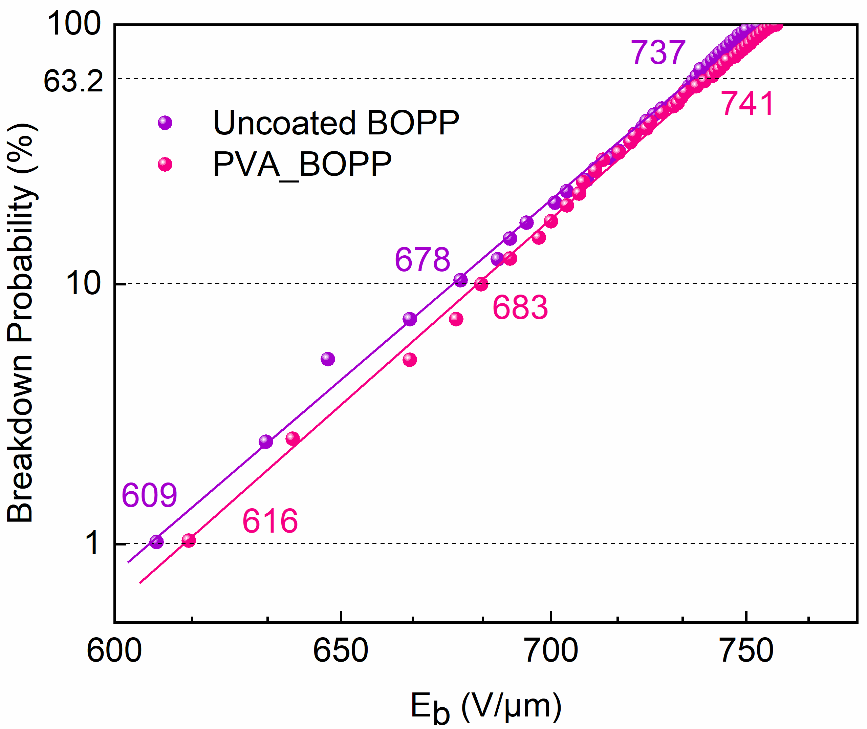
**

**Figure S6.** Weibull distribution of the breakdown strengths of uncoated and PVA-coated BOPP.


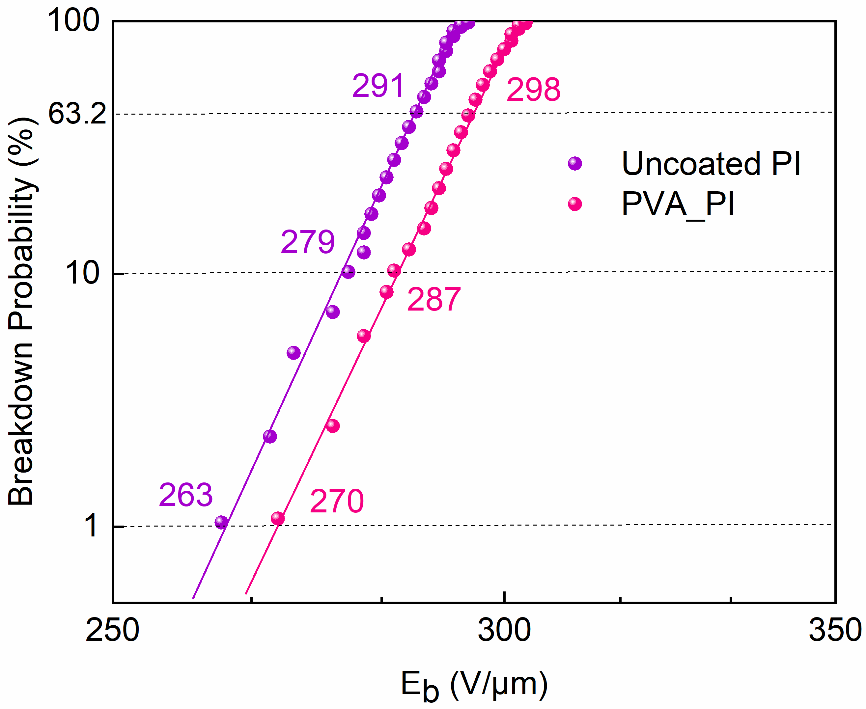


**Figure S7.** Weibull distribution of the breakdown strengths of uncoated and PVA-coated PI.


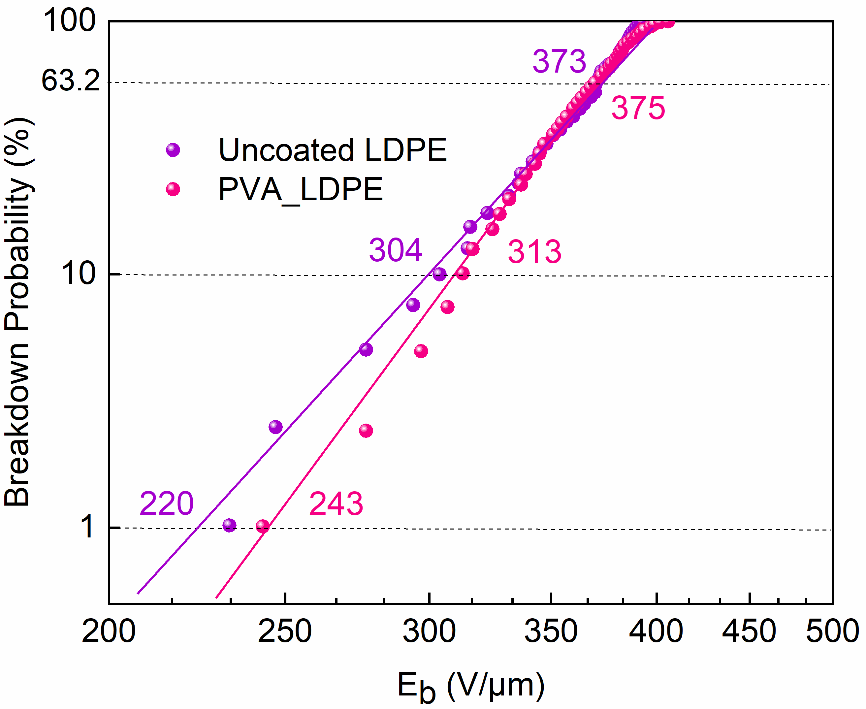


**Figure S8.** Weibull distribution of the breakdown strengths of uncoated and PVA-coated LDPE.

**
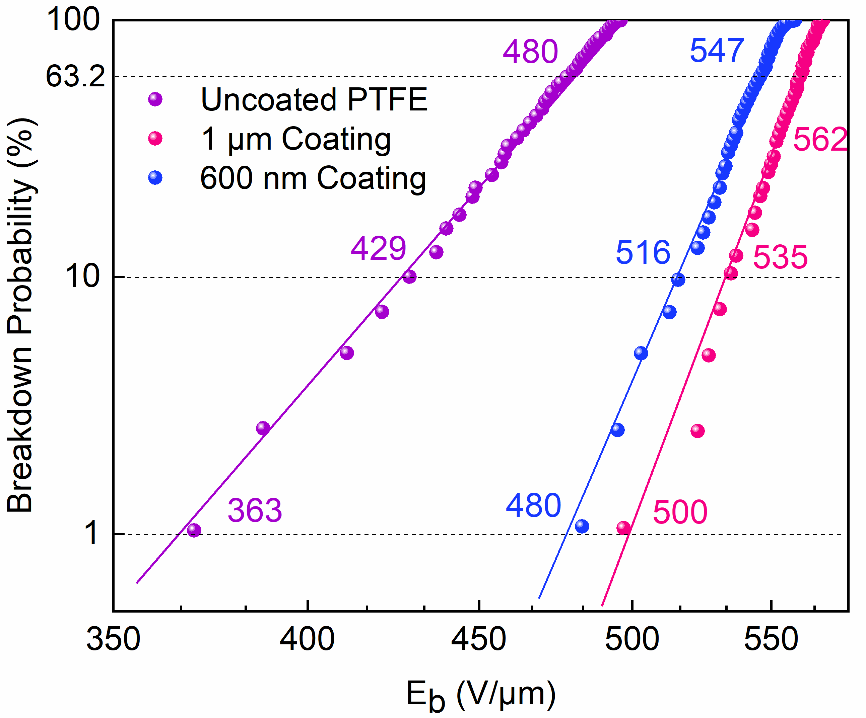
**

**Figure S9.** Weibull distribution of the breakdown strengths of uncoated and coated PTFE with different thicknesses.

**
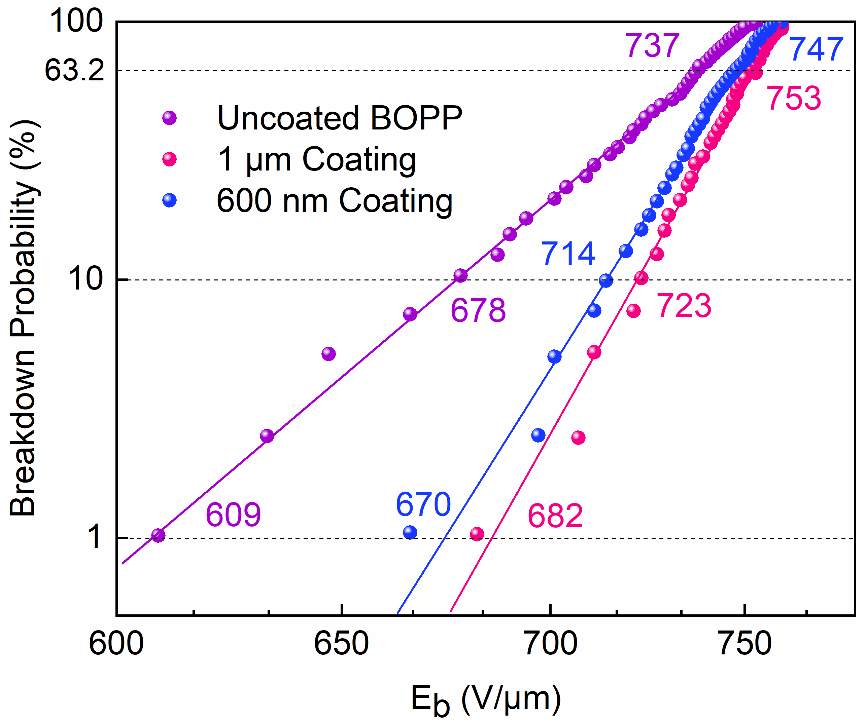
**

**Figure S10.** Weibull distribution of the breakdown strengths of uncoated and coated BOPP with different thicknesses.

**
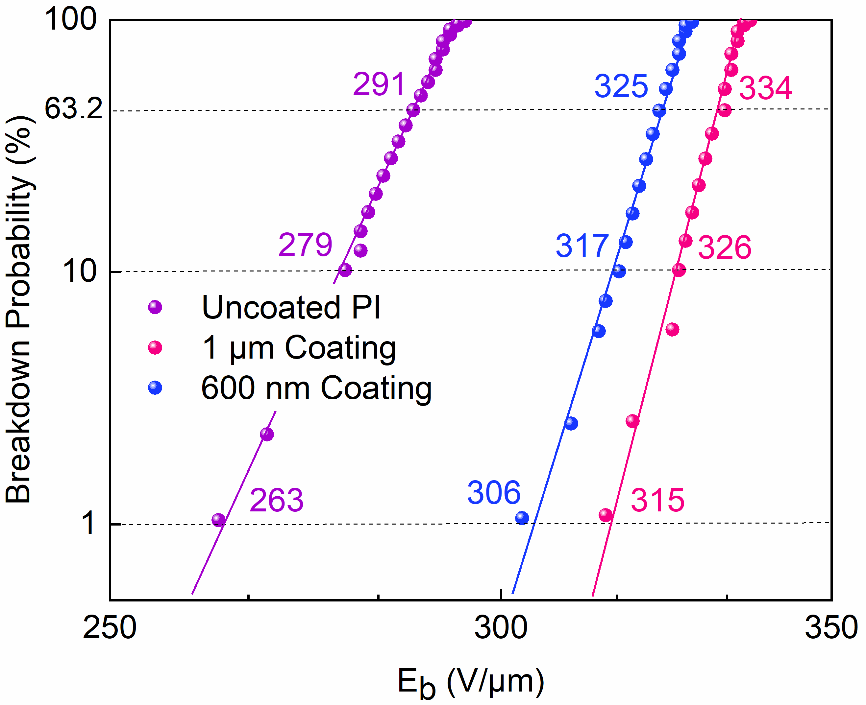
**

**Figure S11.** Weibull distribution of the breakdown strengths of uncoated and coated PI with different thicknesses.

**
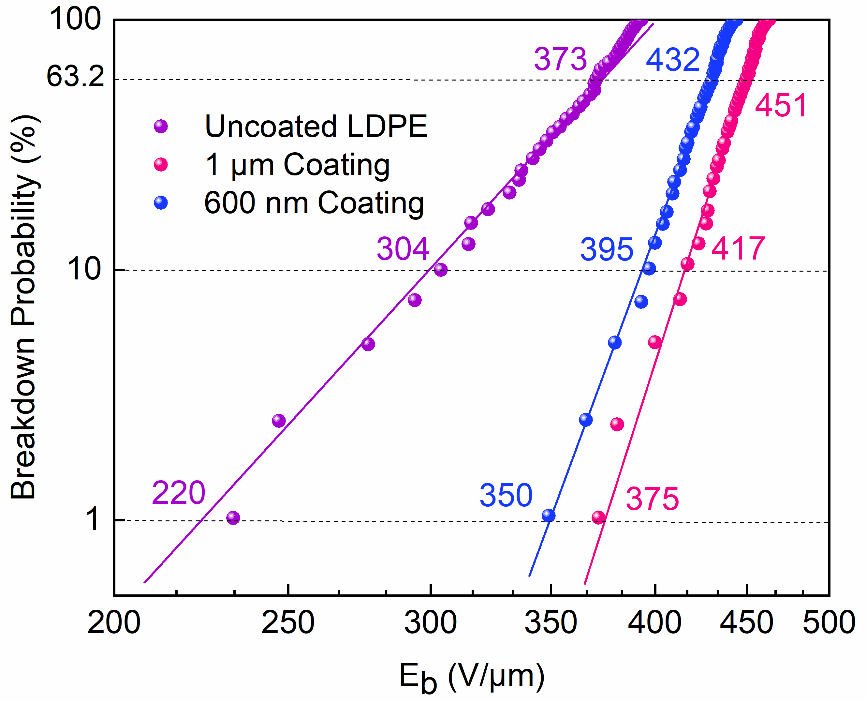
**

**Figure S12.** Weibull distribution of the breakdown strengths of uncoated and coated LDPE with different thicknesses.

**
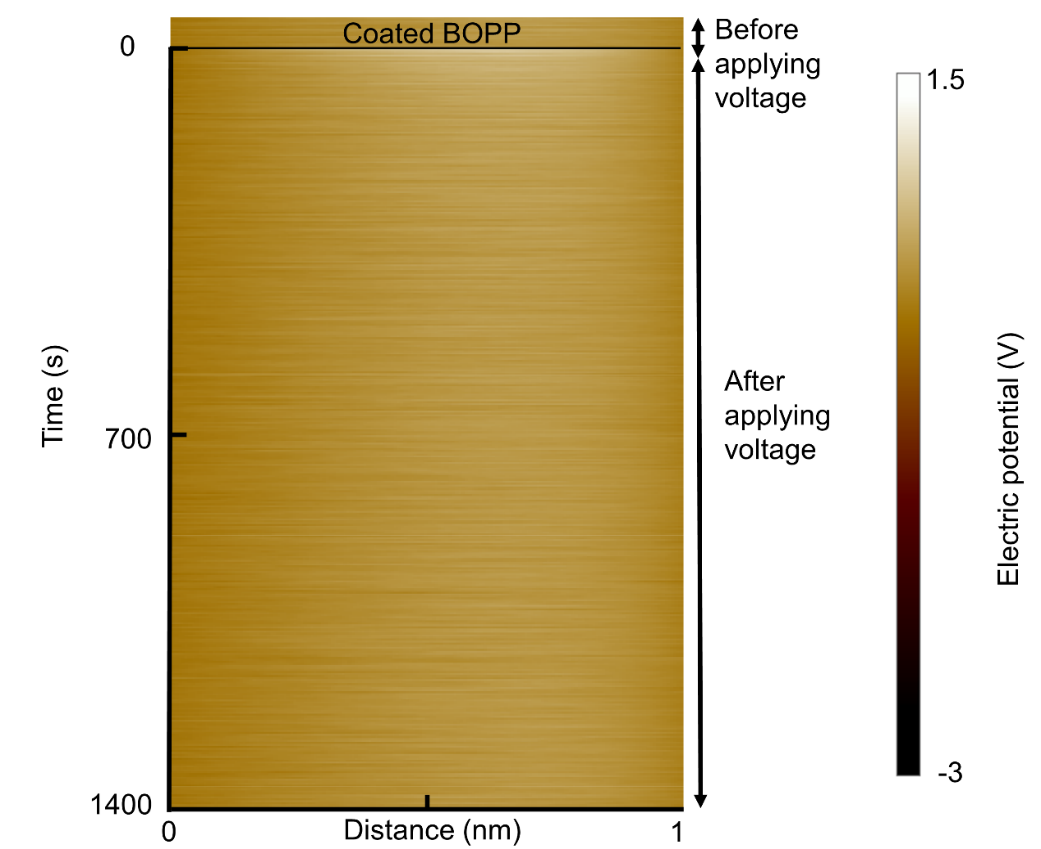
**

**Figure S13.** KPFM-based surface potential dissipation diagram of coated BOPP.

**
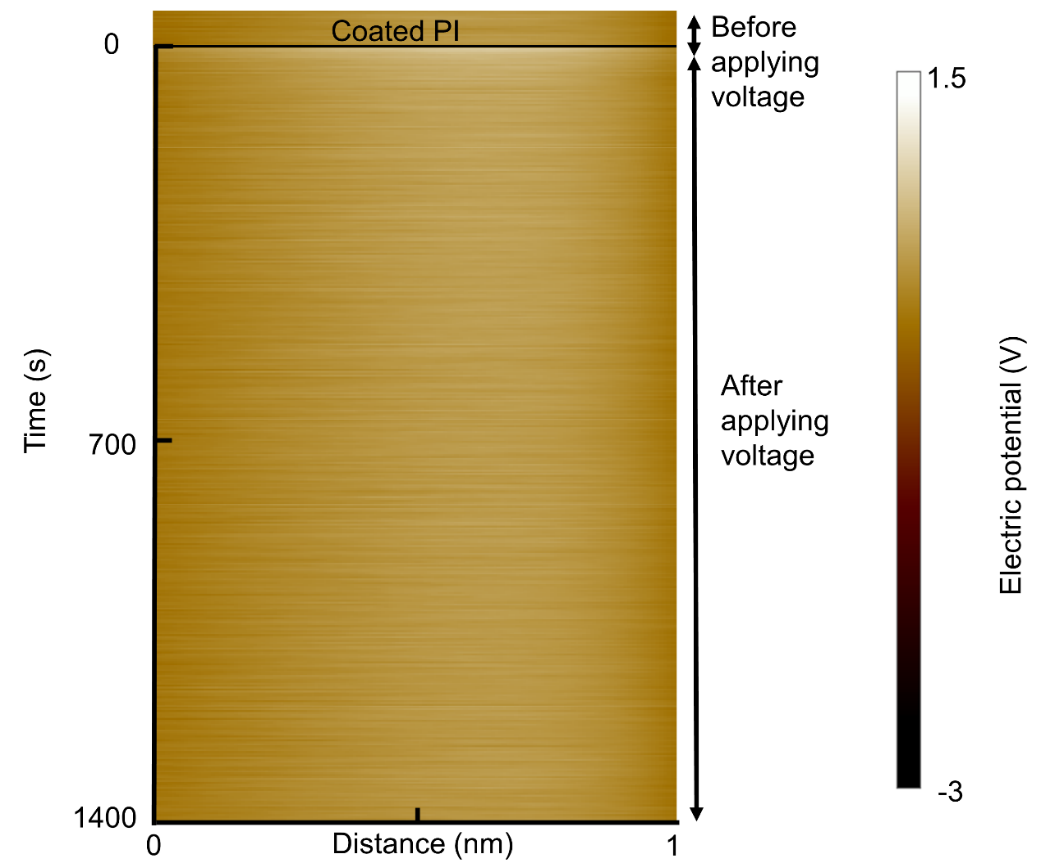
**

**Figure S14.** KPFM-based surface potential dissipation diagram of coated PI.

**
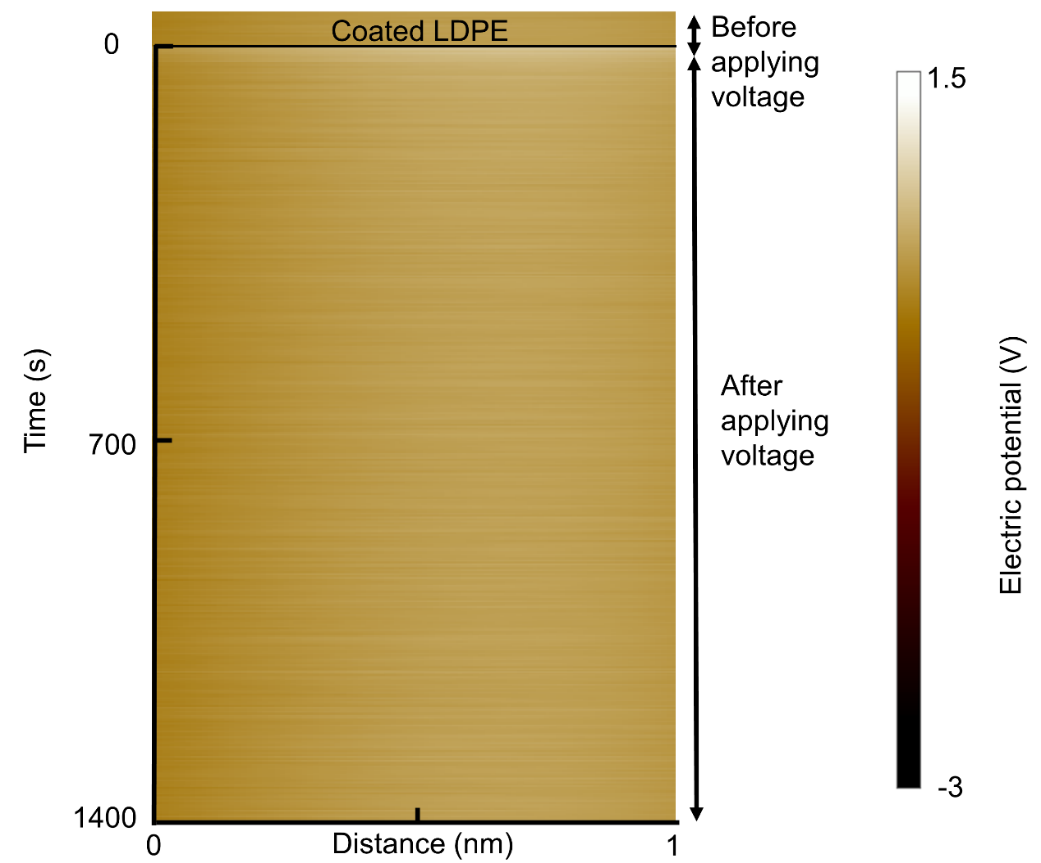
**

**Figure S15.** KPFM-based surface potential dissipation diagram of coated LDPE.

**
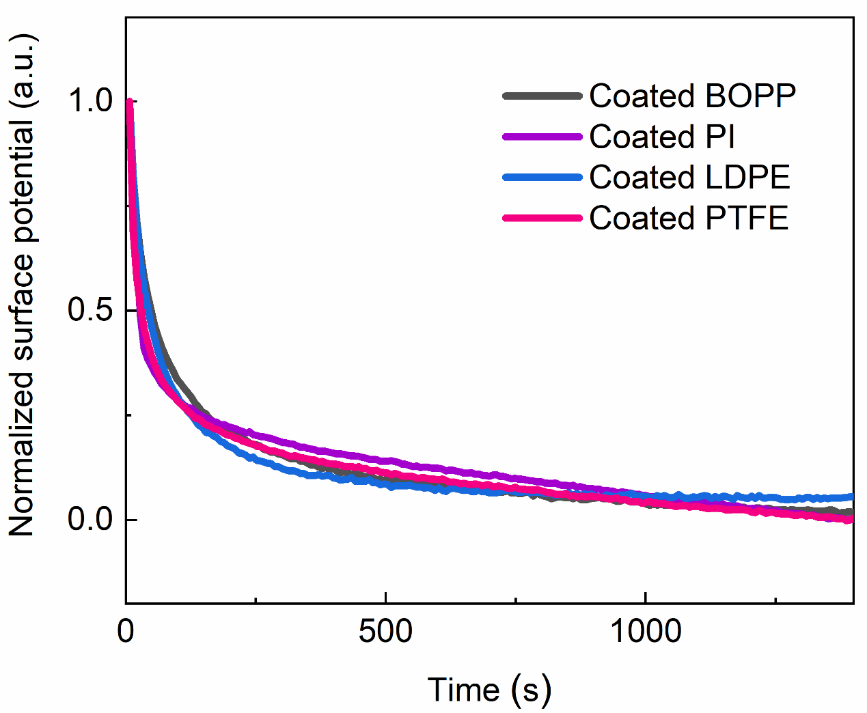
**

**Figure S16.** Normalized surface potential dissipation curves based on KPFM for different kinds of polymer dielectrics after coating.


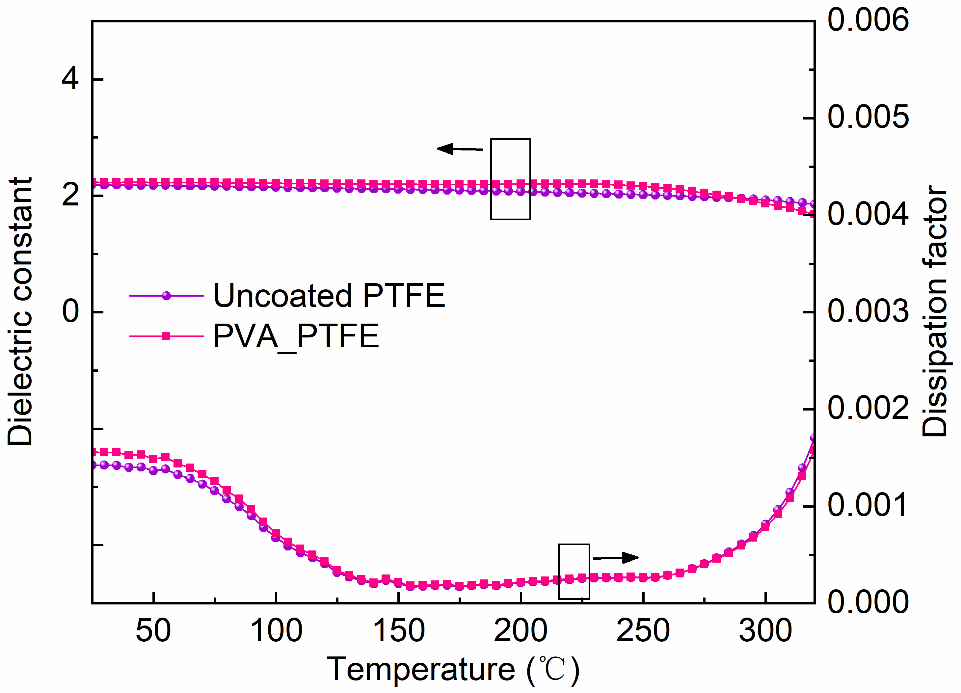


**Figure S17.** K and DF values of uncoated and PVA-coated PTFE.


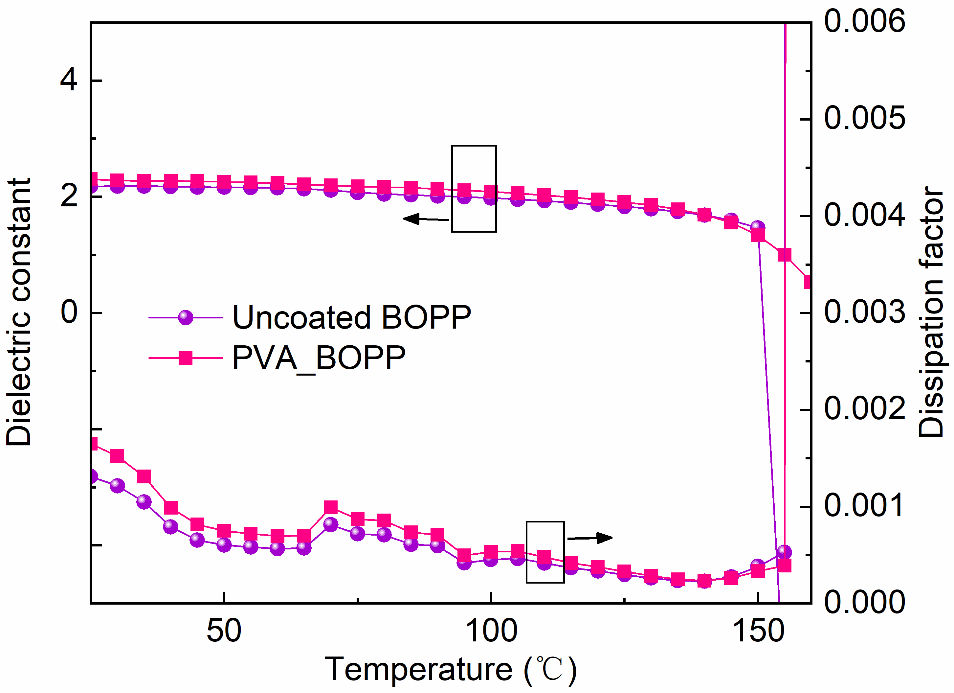


**Figure S18.** K and DF values of uncoated and PVA-coated BOPP.


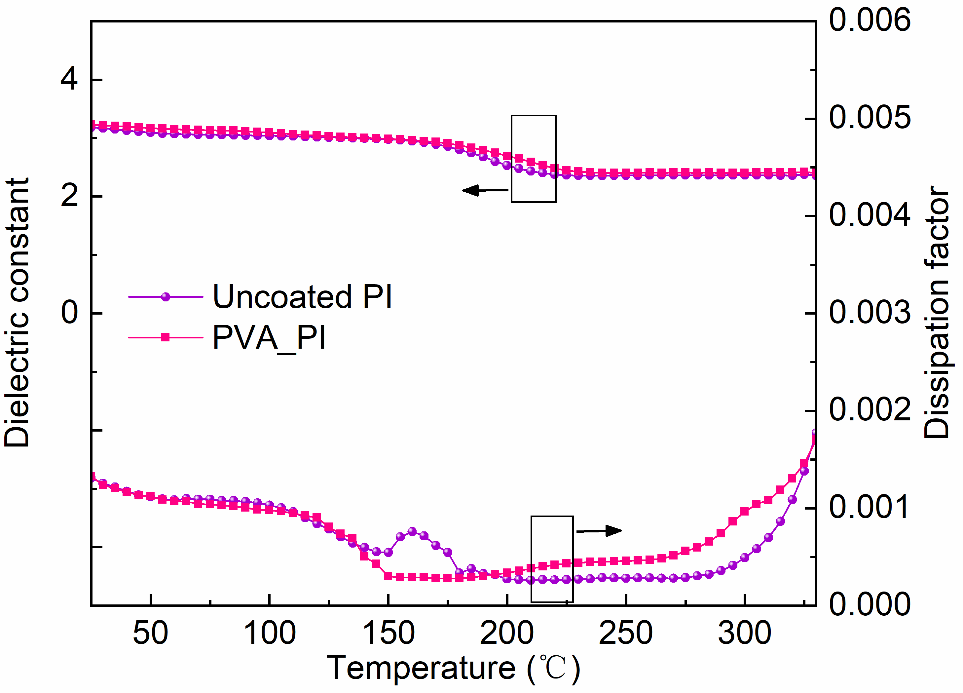


**Figure S19.** K and DF values of uncoated and PVA-coated PI.


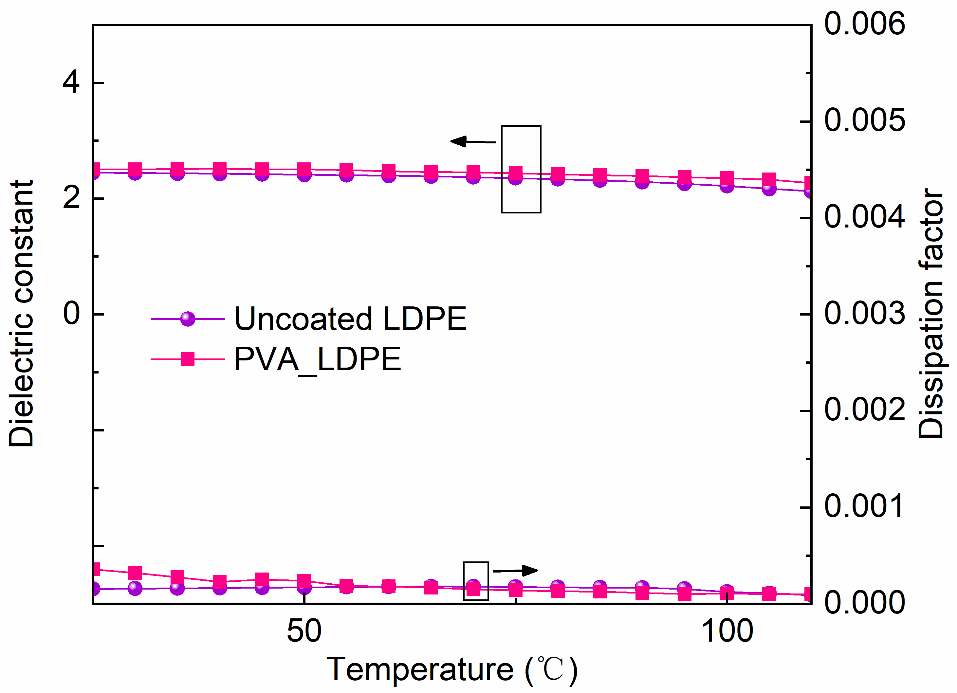


**Figure S20.** K and DF values of uncoated and PVA-coated LDPE.


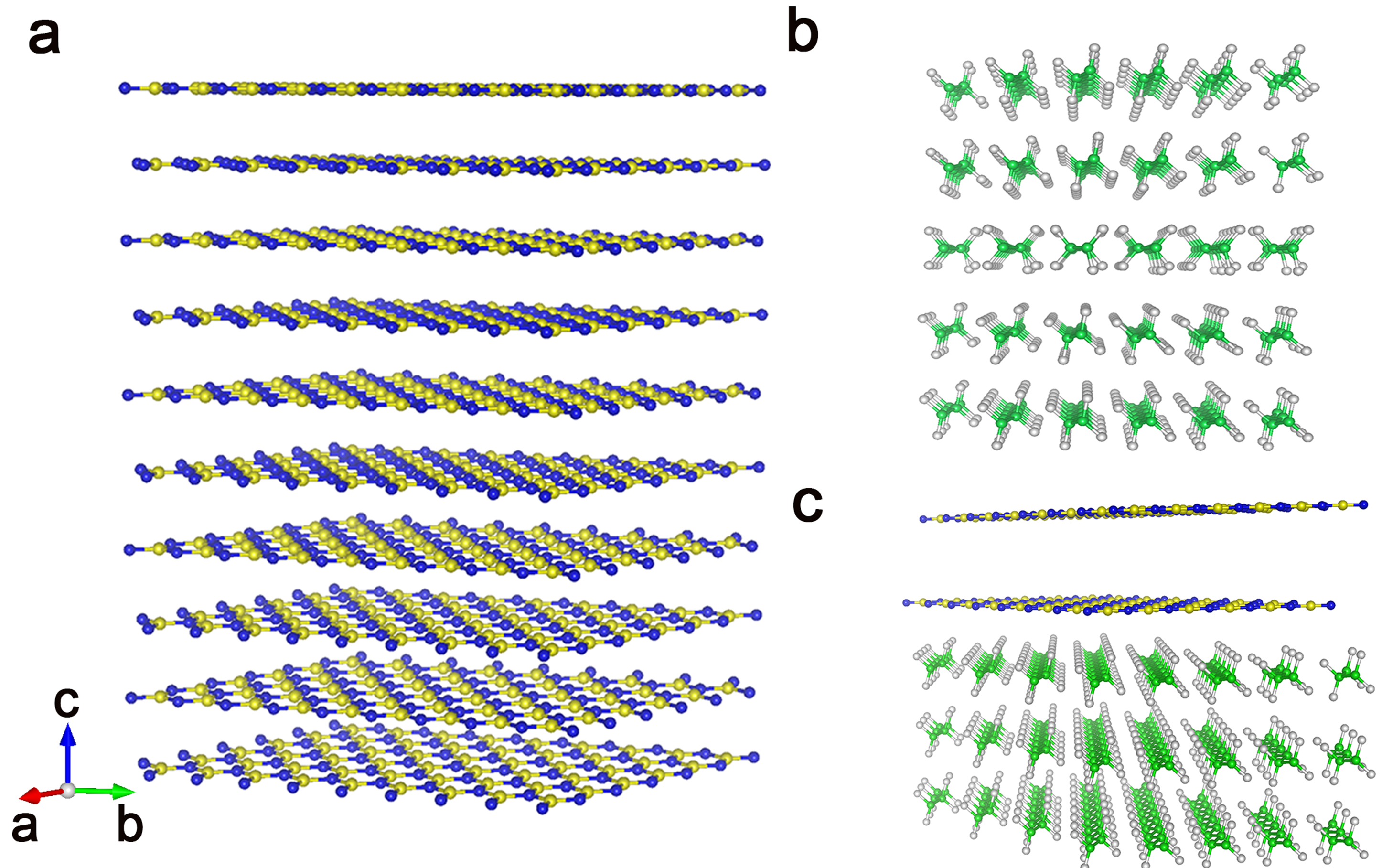


**Figure S21.** Slab prototypes of (a) the (001) surface of BNNS, (b) the (001) surface of PTFE, and (c) BNNS@ PTFE heterojunction. The atoms are represented by spheres: N (blue), B (yellow), C (green), and F (white).


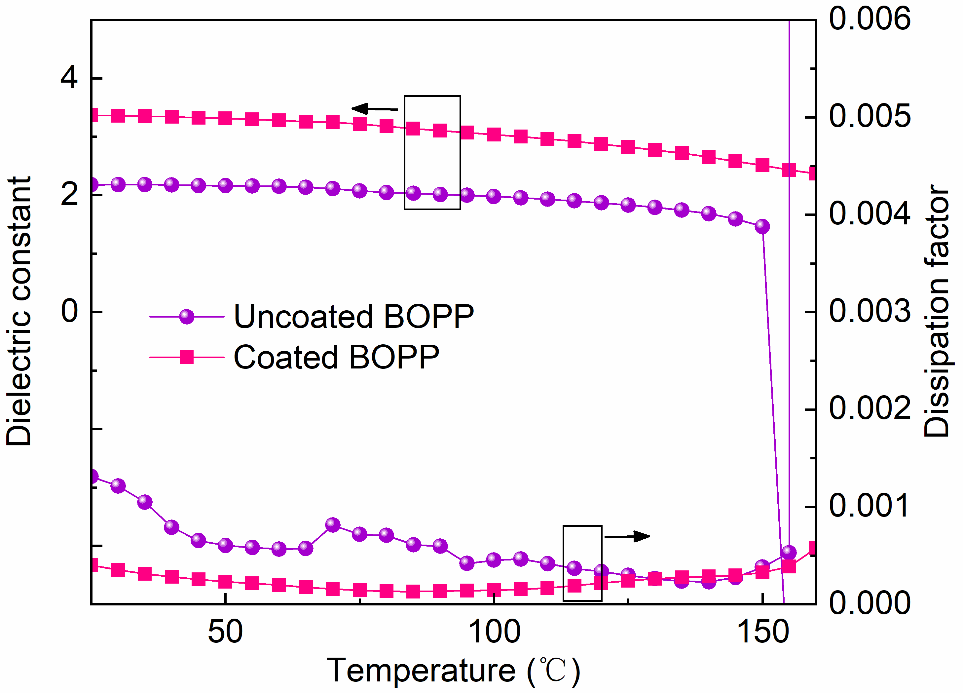


**Figure S22.** Changes in the K and DF values of BOPP with temperature (frequency of 10^4^ Hz) before and after coating.


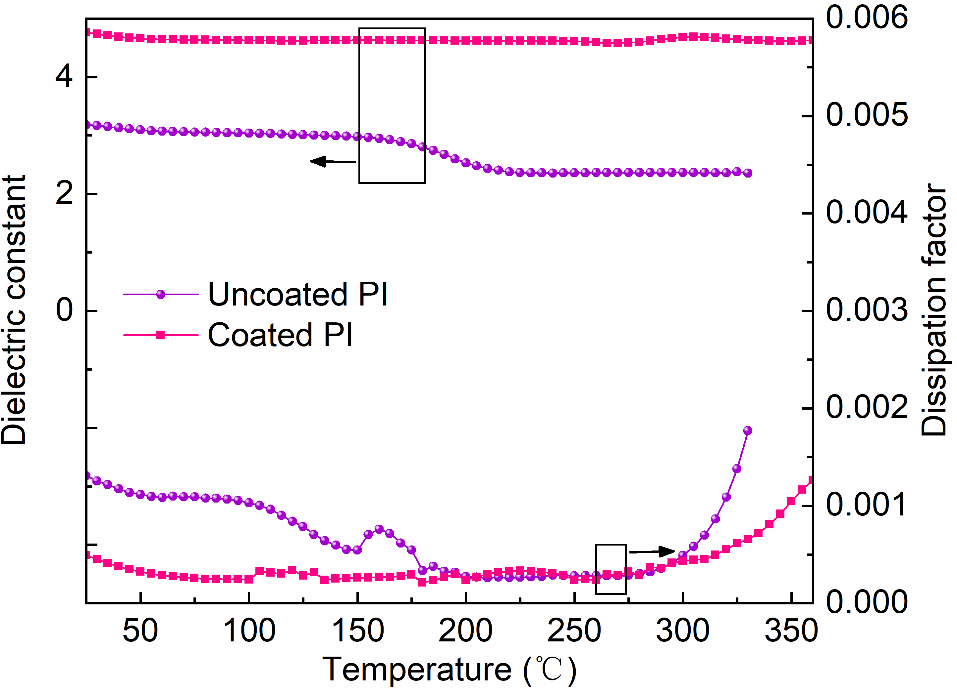


**Figure S23.** Changes in the K and DF values of PI with temperature (frequency of 10^4^ Hz) before and after coating.


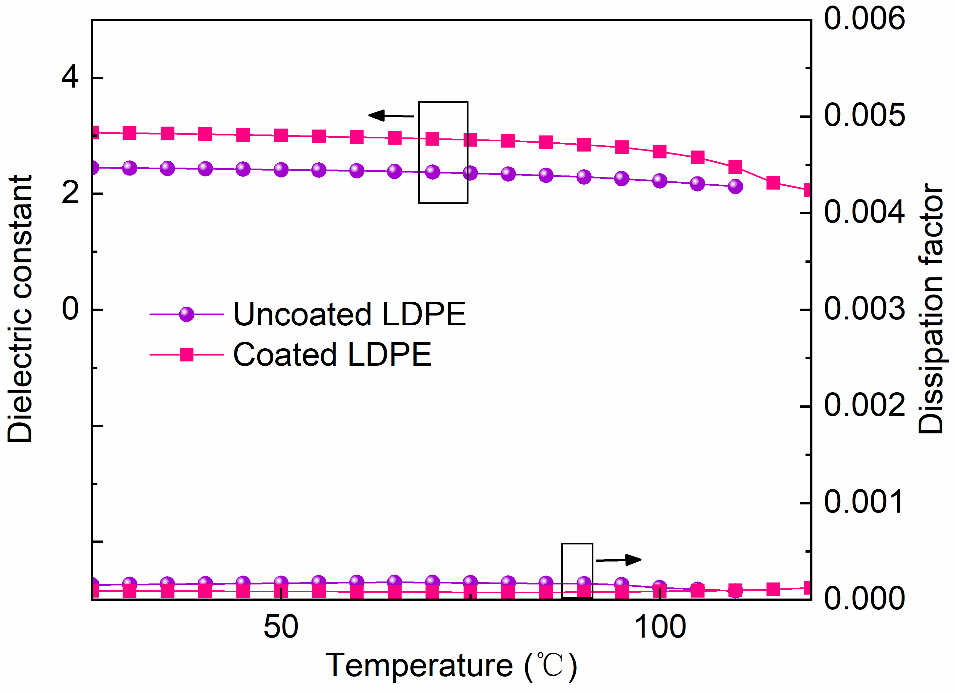


**Figure S24.** Changes in the K and DF values of LDPE with temperature (frequency of 10^4^ Hz) before and after coating.


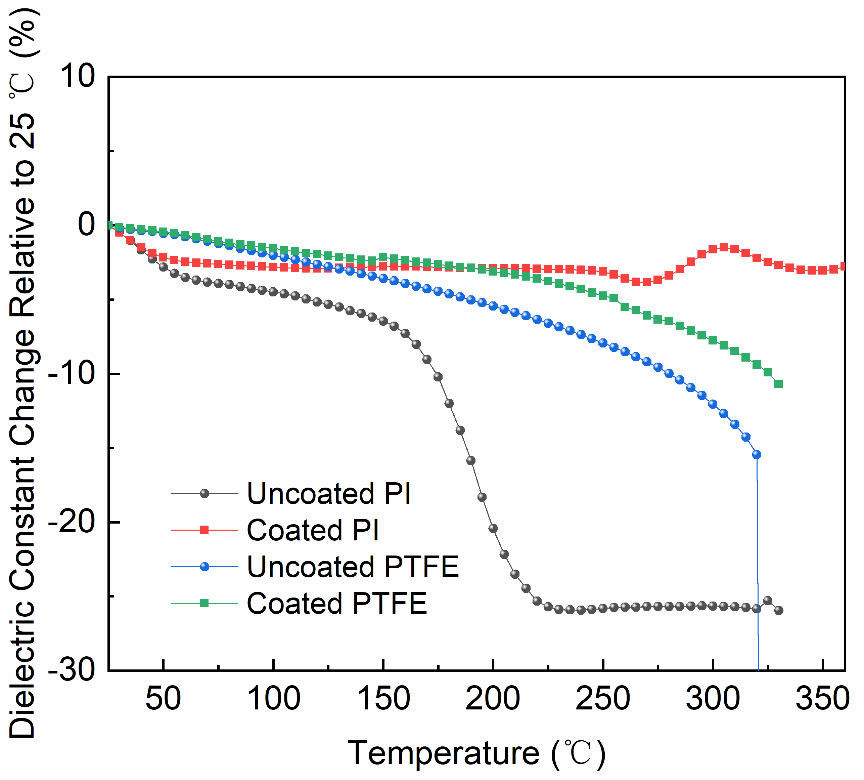


**Figure S25.** Percent change in the K values of PI and PTFE before and after coating at different temperatures relative to those at 25 °C (frequency of 10^4^ Hz).


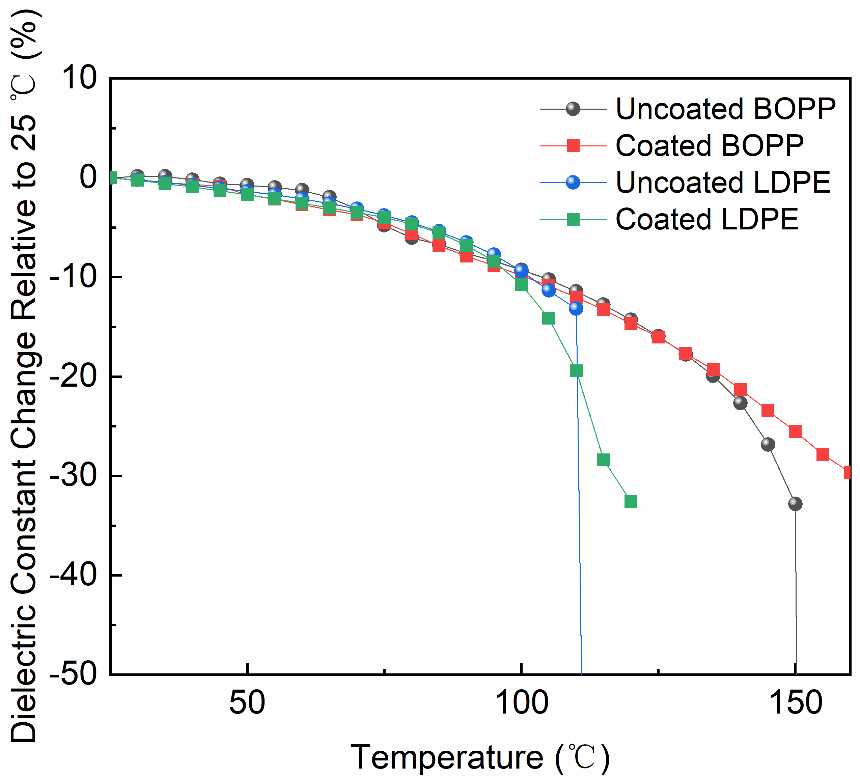


**Figure S26.** Percent change in the K values of BOPP and LDPE before and after coating at different temperatures relative to those at 25 °C (frequency of 10^4^ Hz).


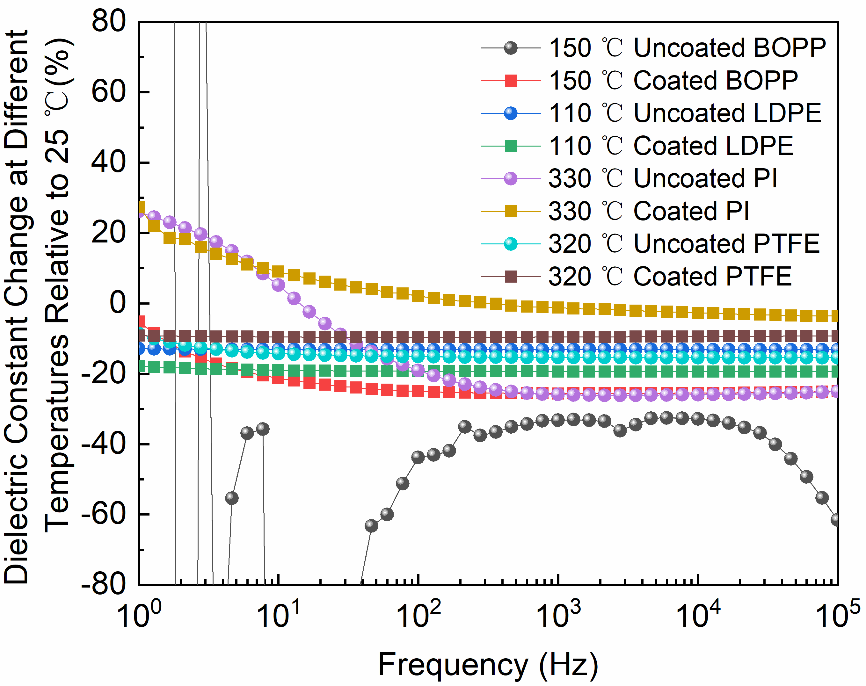


**Figure S27.** Percent change in the K value of polymer dielectrics before and after coating at extreme temperatures relative to 25 °C at different frequencies.


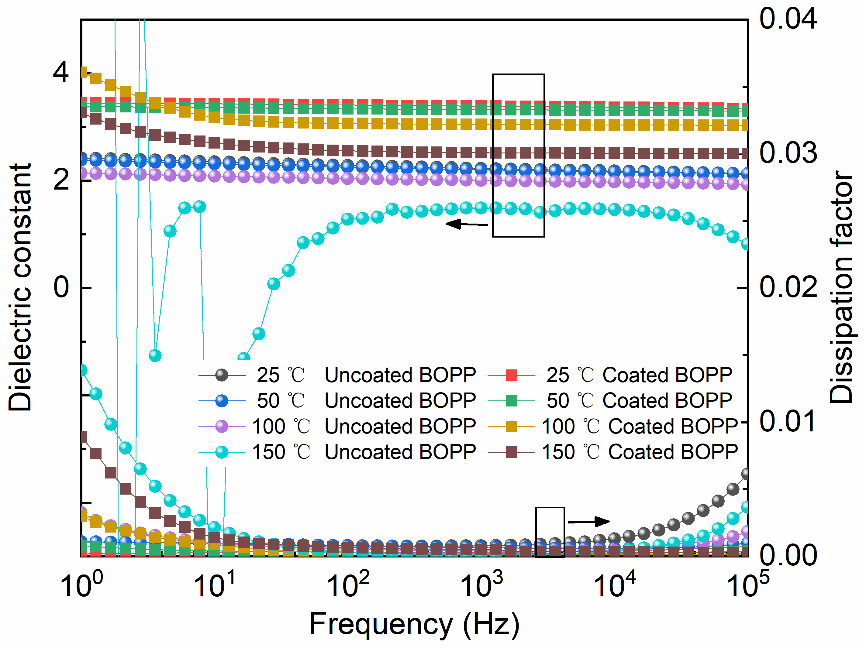


**Figure S28.** Changes in the K and DF values of BOPP with frequency at different temperatures before and after coating.


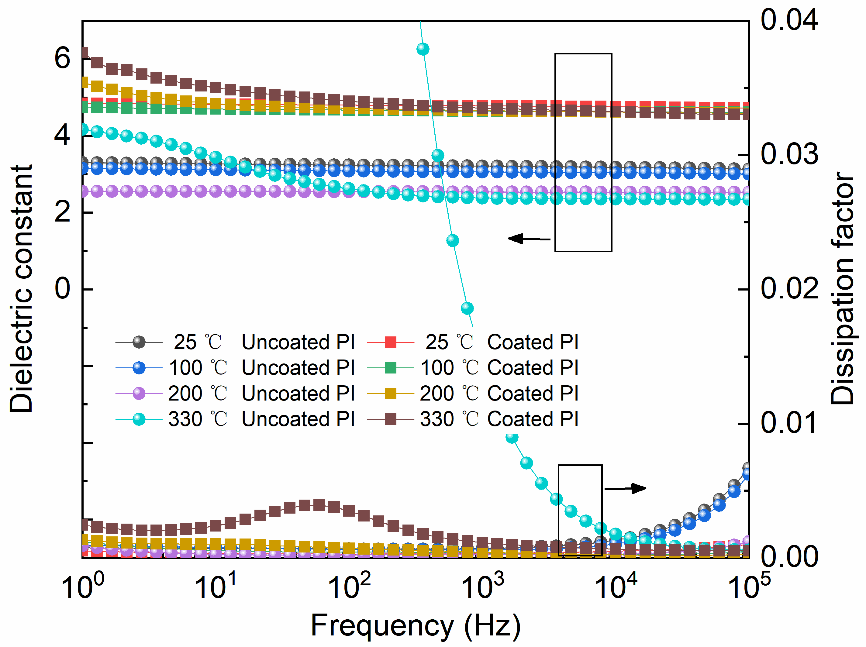


**Figure S29.** Changes in the K and DF values of PI with frequency at different temperatures before and after coating.


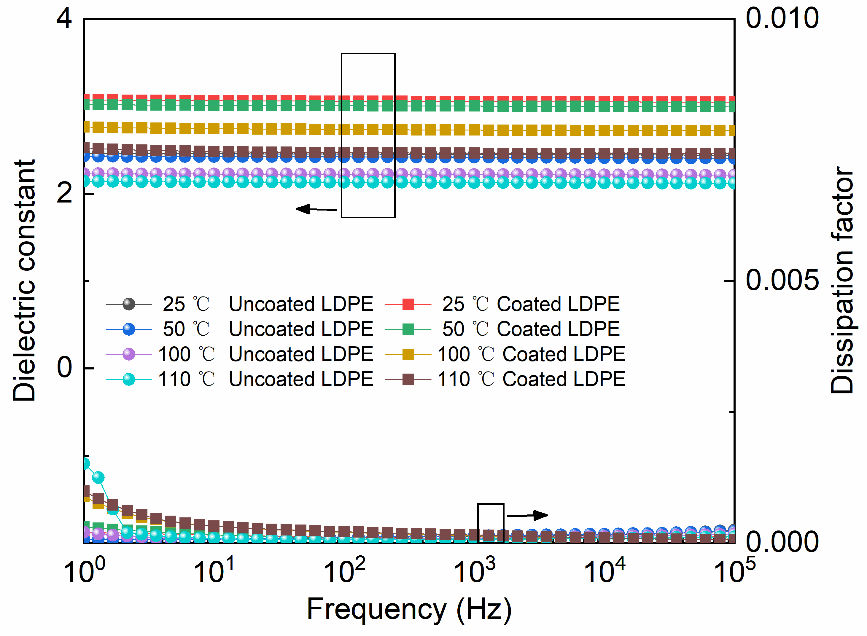


**Figure S30.** Changes in the K and DF values of LDPE with frequency at different temperatures before and after coating.


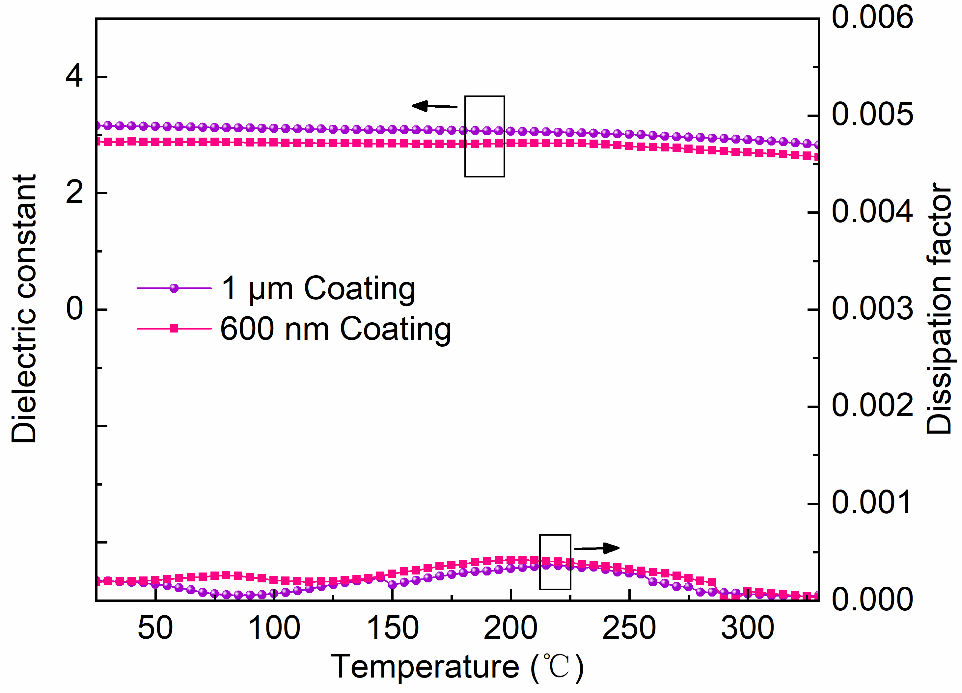


**Figure S31.** K and DF values of PTFE coated with films of different thicknesses as a function of temperature (frequency of 10^4^ Hz).


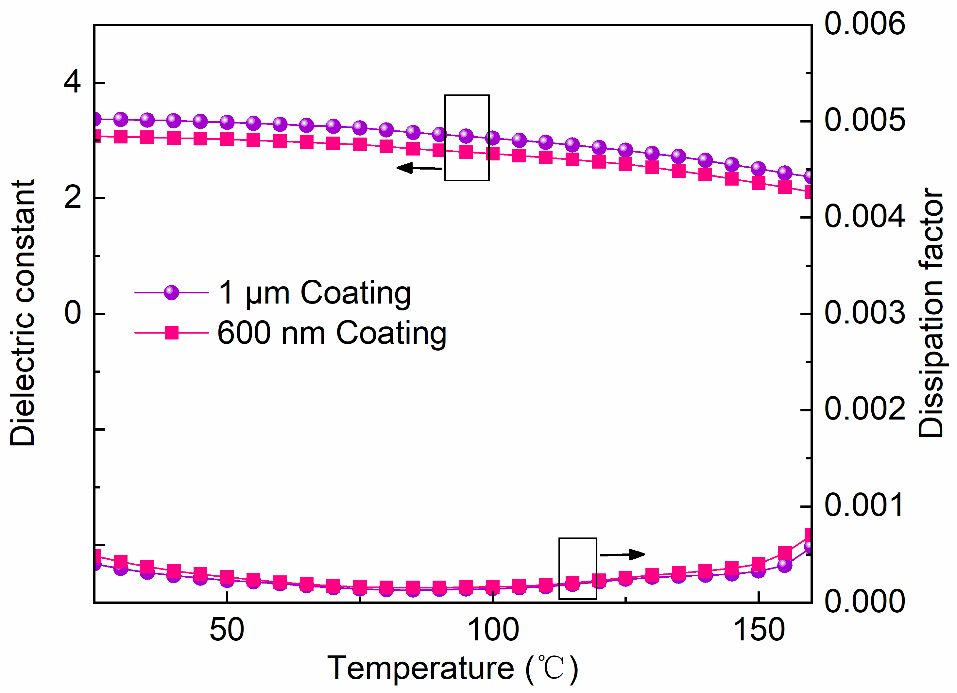


**Figure S32.** K and DF values of BOPP coated with films of different thicknesses as a function of temperature (frequency of 10^4^ Hz).


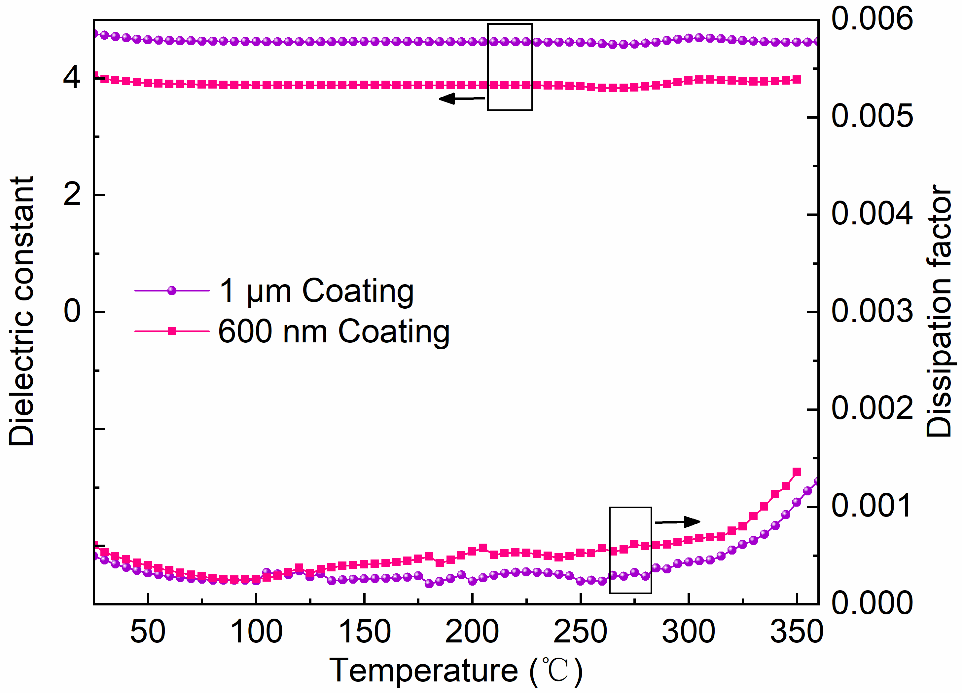


**Figure S33.** K and DF values of PI coated with films of different thicknesses as a function of temperature (frequency of 10^4^ Hz).


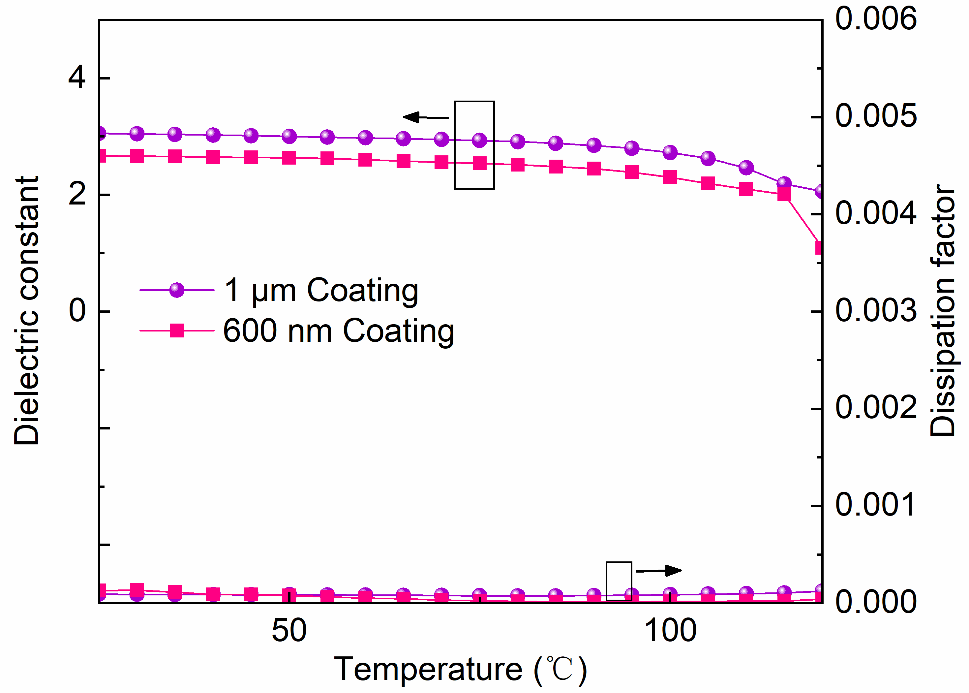


**Figure S34.** K and DF values of LDPE coated with films of different thicknesses as a function of temperature (frequency of 10^4^ Hz).

| Samples | 1% breakdown probability | 10% breakdown probability | 63.2% breakdown probability | Shape coefficient β |
| --- | --- | --- | --- | --- |
| Uncoated PTFE | 363 | 429 | 480 | 20 |
| 1 μm coated PTFE | 500 | 535 | 562 | 45 |
| Increase | 37.7% | 24.7% | 17% | 125% |
| 600 nm coated PTFE | 480 | 516 | 547 | 39 |
| Increase | 32.2% | 20.2% | 13.9% | 95% |
| PVA-coated PTFE | 383 | 437 | 483 | 22.5 |
| Increase | 5.5% | 1.8% | 0.6% | 12.5% |
| Uncoated BOPP | 609 | 678 | 737 | 27 |
| 1 μm coated BOPP | 682 | 723 | 753 | 55 |
| Increase | 11.9% | 6.6% | 2.1% | 103% |
| 600 nm coated BOPP | 670 | 714 | 747 | 50 |
| Increase | 10% | 5.3% | 1.4% | 85% |
| PVA-coated BOPP | 616 | 683 | 741 | 27.6 |
| Increase | 1.1% | 0.7% | 0.5% | 2.2% |
| Uncoated PI | 263 | 279 | 291 | 53 |
| 1 μm coated PI | 315 | 326 | 334 | 92 |
| Increase | 19.7% | 16.8% | 14.7% | 73% |
| 600 nm coated PI | 306 | 317 | 325 | 90 |
| Increase | 16.3% | 13.6% | 11.7% | 69.8% |
| PVA-coated PI | 270 | 287 | 298 | 59 |
| Increase | 2.6% | 2.8% | 2.4% | 11.3% |
| Uncoated LDPE | 220 | 304 | 373 | 11 |
| 1 μm coated LDPE | 375 | 417 | 451 | 28 |
| Increase | 70% | 37% | 21% | 154% |
| 600 nm coated LDPE | 350 | 395 | 432 | 25 |
| Increase | 59.1% | 29.9% | 15.8% | 127% |
| PVA-coated LDPE | 243 | 313 | 375 | 12.4 |
| Increase | 10.4% | 2.9% | 0.5% | 12.7% |

**Table S1.** Breakdown Strength Weibull Analysis Detailed Numerical Values

**Table S2.** Static dielectric constants ε*_II_*, ε*_JJ_*, and ε*_KK_* in three principal directions and the average static dielectric constant ε_av_ of the (001) surface of BNNS, the (001) surface of PTFE, and the BNNS@PTFE heterojunction.

| Dielectric Constant | (001) surface of BNNS | (001) surface of PTFE | BNNS@PTFE heterojunction |
| --- | --- | --- | --- |
| ε*_II_* | 2.95 | 1.56 | 2.96 |
| ε*_JJ_* | 2.92 | 1.69 | 1.94 |
| ε*_KK_* | 1.46 | 1.35 | 1.44 |
| ε_av_ | 2.44 | 1.53 | 2.11 |
